# Wuzhuyu Decoction Relieves Chronic Migraine by Regulating 5-HT1A and 3A Receptors-Mediated CREB Signaling Pathway in Brain and Intestine

**DOI:** 10.3390/ph18030426

**Published:** 2025-03-18

**Authors:** Zhimin Song, Meijing Li, Ziwei Zhou, Xiaomeng Guo, Qi Wang, Zekuan Zhang, Keshu Wang, Qixiang Zheng, Wenjing Gou, Sha Wu, Hui Zhao, Muxin Gong

**Affiliations:** 1 School of Traditional Chinese Medicine, Capital Medical University, No. 10 of Xitoutiao, Youanmenwai, Fengtai District, Beijing 100069, China; songzhimin00@163.com (Z.S.); lmj15810192085@163.com (M.L.); zhouziwei199509@163.com (Z.Z.); gxmnzyx@163.com (X.G.); 112021020125@mail.ccmu.edu.cn (Q.W.); zhangzekuan0628@163.com (Z.Z.); 15768982081@163.com (K.W.); zhengqixiang2001@163.com (Q.Z.); gouwenjing0416@163.com (W.G.); wusha729@163.com (S.W.); zhaohui8957@sina.com (H.Z.); 2 Beijing Key Laboratory of Traditional Chinese Medicine Collateral Disease Theory Research, Beijing 100069, China

**Keywords:** Wuzhuyu decoction, chronic migraine, pain sensitization, 5-HT1A receptor, 5-HT3A receptor, CREB signaling pathway

## Abstract

**Background:** Chronic migraine (CM) is a common complex nervous system disease, often accompanied by symptoms of the digestive tract that interact with each other, leading to prolonged and difficult-to-cure migraines. These symptoms are associated with abnormalities in 5-HT and its receptors. Wuzhuyu decoction (WZYD) is a traditional Chinese medicine prescription commonly used in clinics to treat CM; it relieves gastrointestinal symptoms, such as nausea and vomiting; however, its mechanism is still unclear. Investigating the differences in the role of WZYD compared to existing drugs targeting 5-HT receptors in the treatment of CM not only helps elucidate its pathogenesis but also provides possibilities for the development of new therapeutic approaches. **Methods:** An inflammation soup (IS)-induced CM male rat model was established. Based on a preliminary experiment, the target of WZYD in treating CM was determined by network pharmacology, and verified by molecular docking. ELISA, immunofluorescence, western blot, and real-time quantitative polymerase chain reaction (RT-qPCR) were used to evaluate the expression levels of CM-related indicators (5-HT, calcitonin gene-related peptide (CGRP), and c-Fos) to ensure the successful establishment of the CM model and the effectiveness of the drug. On this basis, the protein expression levels of 5-HT1A/3A receptors and their cAMP-response element binding protein (CREB) signaling pathway were detected by western blot and immunohistochemistry. The role of 5-HT1A/3A receptors in the treatment of CM by WZYD was validated using a 5-HT1A receptor antagonist (WAY 100635) and a 5-HT3A receptor agonist (SR 57227). **Results:** The results showed that WZYD increased the expression of 5-HT in the brain, decreased the expression of CGRP, c-Fos, ionized calcium-binding adapter molecule 1 (Iba1), and relieved CM. At the same time, WZYD also increased the expression of the 5-HT1A receptor and decreased the expression of the 5-HT3A receptor in the brain and colon of CM rats. Subsequently, WZYD further exerted its brain-gut integrated therapeutic effects by regulating the CREB signaling pathway mediated by 5-HT1A/3A receptors in the brain and colon of CM rats. **Conclusions:** WZYD not only regulates neurotransmitters in the brain and colon at the same time, but also specifically regulates 5-HT1A/3A receptors in the brain and colon, which explains the characteristics and advantages of WZYD from a new perspective. While effectively relieving headache symptoms, it also improves related gastrointestinal symptoms, which is more conducive to the treatment of CM.

## 1. Introduction

Chronic migraine (CM) is a type of recurrent brain dysfunction disorder influenced by both genetic and environmental factors. It usually develops from migraine and is characterized by moderate to severe unilateral pulsating headache, often accompanied by symptoms of the digestive tract [1,2]. The quality of life of patients with CM is seriously affected. At present, the analgesic medicines used for migraine are mostly single-target drugs, mainly to relieve headache symptoms [3]. Ergotamine, flunarizine, atogepant, and botulinum toxin A are commonly used medicines in the clinical treatment of CM, which not only have limited efficacy but are also prone to adverse reactions, such as medication-overuse headache and gastrointestinal reactions after long-term use [4,5]. The therapeutic options for CM remain limited.

Wuzhuyu decoction (WZYD), a traditional Chinese polyherbal formulation originating from Zhang Zhongjing’s “Shang Han Lun”, is mainly used for headache, vomiting, abdominal pain, and other diseases [6]. Clinical studies suggest that WZYD has great potential to improve CM. In a randomized, double-blind, placebo-controlled trial, the efficacy of WZYD granules in treating migraine patients was investigated with 12 weeks of administration. The results indicated that WZYD granules could effectively reduce the frequency and number of days of migraine attacks, and the treatment was well tolerated and safe [7]. In addition, clinical case analysis of WZYD showed no severe toxicity or adverse reactions in patients who took WZYD [8,9]. The experimental research indicates that WZYD’s treatment of CM exhibits multi-target and multi-pathway characteristics, not only alleviating headaches but also regulating intestinal functions [10,11]. 5-HT plays an important role in the pathogenesis of migraine, and its function depends on the activation of 5-HT receptors. After activation, 5-HT receptors further regulate downstream signaling pathways and participate in the regulation of pain and gastrointestinal symptoms. The 5-HT receptor family consists of seven types and 14 subtypes distributed in both central and peripheral tissues. The activation of different 5-HT receptors in different tissues has different effects, and some 5-HT receptors play an important role in regulating central pain and peripheral gastrointestinal functions [12,13]. CM is often accompanied by symptoms such as nausea and vomiting, and CM is significantly related to gastrointestinal diseases such as irritable bowel syndrome and functional dyspepsia [14,15]. Nausea, vomiting, and gastrointestinal diseases are often related to 5-HT and 5-HT receptors. While treating CM, WZYD often improves gastrointestinal symptoms, which may be related to its regulation of 5-HT and the specific modulation of 5-HT receptors.

Previous research has found that there is a complex interaction between intestinal flora and migraine, and an imbalance in intestinal flora may lead to the impairment of intestinal barrier function, thus inducing migraine. However, these studies only focused on changes in intestinal flora and did not determine the role of 5-HT receptors between the brain and gastrointestinal tract [16]. Considering that 5-HT receptors are involved in the pathogenesis of various nervous system and gastrointestinal diseases, it is of great clinical significance to study the effect of WZYD on improving CM and its related gastrointestinal symptoms by regulating 5-HT receptors in the brain and intestine, which will further clarify the mechanism and therapeutic characteristics of WZYD. However, research reports in this field are still lacking.

The existing drugs for treating CM with 5-HT receptors mainly focus on 5-HT 1B/1D/1F receptors, and these drugs have serious side effects [4]. At present, there is no research on treating migraine with 5-HT1A/3A receptors as the target from the perspective of brain-gut co-treatment. Before the formal experiment, we conducted a preliminary experiment. We found that among the many 5-HT receptors, WZYD had little effect on the expression of 5-HT1B/1D/1F/2A/7 receptors but specifically regulated the expression of 5-HT1A and 5-HT3A receptors. The 5-HT1A and 5-HT3A receptors play important roles in the modulation of pain and gastrointestinal symptoms. Activation of the 5-HT1A receptor inhibits the excessive excitation of neurons, reduces the release of neurotransmitters related to pain, and thus alleviates pain [17]. 5-HT1A receptor is also involved in regulating the function of the gastrointestinal tract, and its agonists are used to treat many diseases in the clinic, such as functional dyspepsia and irritable bowel syndrome [18]. In contrast, activation of the 5-HT3A receptor increase sneuronal excitability and regulates the release of neurotransmitters, thereby enhancing pain perception. The 5-HT3A receptor is highly expressed in the myenteric and submucosal plexuses of the gastrointestinal tract. It can increase the excitability of enteric neurons and promote the release of neurotransmitters, thereby enhancing gastrointestinal motility [19,20]. For example, 5-HT3A receptor antagonists (such as tropisetron and ondansetron) are commonly used to relieve nausea and vomiting caused by chemotherapy [21]. For diseases like CM, where there is mutual interference between the brain and the gut, it is necessary to treat both the brain and the gut simultaneously to avoid their mutual interference. Monotherapy can lead to recurrent symptoms. Therefore, we speculate that WZYD not only regulates 5-HT in both the brain and colon simultaneously but also specifically modulates the 5-HT1A/3A receptors in the brain and colon.

This study focused on the specific regulatory effect of WZYD on the 5-HT1A/3A receptor while regulating 5-HT and verified the role of the receptor in this process by using receptor agonists and antagonists to clarify the mechanism of brain-intestine cooperative treatment of CM. By inhibiting the central sensitization of pain, improving gastrointestinal symptoms, blocking the interference between the brain and intestine, and realizing more accurate treatment, the characteristics and advantages of WZYD are explained from a new angle.

## 2. Results

### 2.1. Effects of WZYD on Pain Threshold of CM Rats

**Figure 1:**
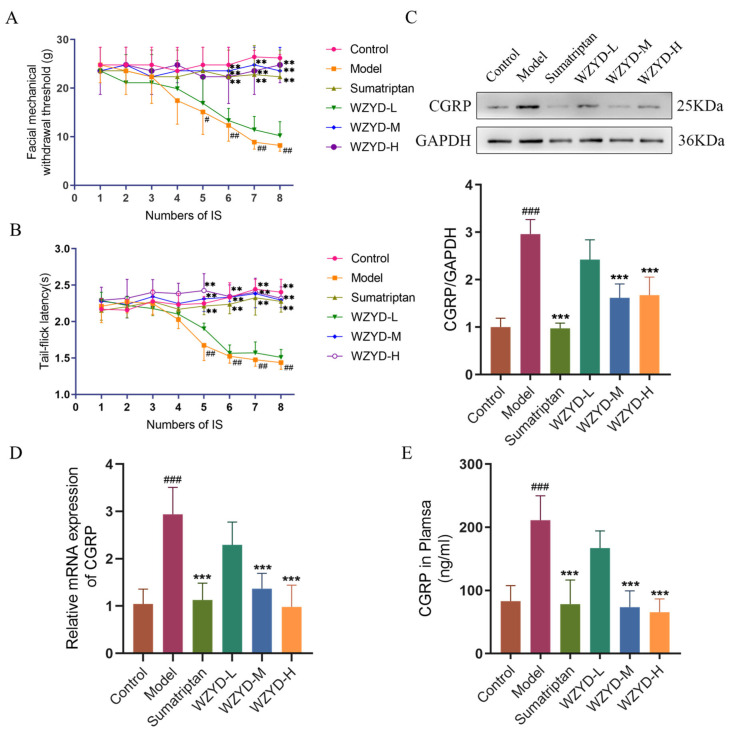
Effects of WZYD on pain threshold and CGRP levels in CM rats. (**A**) Facial mechanical withdrawal threshold (*n* = 9). (**B**) Tail-flick latency (*n* = 9). (**C**) Relative protein expression of CGRP (*n* = 5). (**D**) Relative mRNA expression of CGRP (*n* = 6). (**E**) The expression levels of CGRP in the plasma were measured by ELISA (*n* = 6). Data are expressed as the mean ± SD. ^#^ *p* < 0.05, ^##^ *p* < 0.01, ^###^ *p* < 0.001, vs. the control group; ** *p* < 0.01, *** *p* < 0.001, vs. the model group.

As shown in Figure 1A, the threshold of orbital mechanical pain in the model group decreased after repeated inflammatory soup (IS) stimulation was applied. After the fourth IS stimulation, the threshold of orbital mechanical pain in the model group was significantly lower than that in the control group (*p* < 0.05). After the fifth IS stimulation, compared with the model group, the orbital mechanical pain thresholds in the sumatriptan group (*p* < 0.01), WZYD-M group (*p* < 0.01), and WZYD-H group (*p* < 0.01) were significantly increased.

As shown in Figure 1B, after the fourth IS stimulation, compared with the control group, the tail-flick latencies of the model group rats were significantly reduced (*p* < 0.01); compared with the model group, the tail-flick latencies of the sumatriptan group (*p* < 0.01), WZYD-M group (*p* < 0.01), and WZYD-H group (*p* < 0.01) were all significantly increased after medication.

### 2.2. Effects of WZYD on CGRP in CM Rats

Calcitonin gene-related peptide (CGRP) was a key marker of migraine. The expression levels of CGRP in the plasma and trigeminal nucleus caudate (TNC) were detected (Figure 1C–E). The results demonstrated that the expression of CGRP was significantly increased in the model group. Compared with that in the model group, the content of CGRP in the WZYD-M and WZYD-H groups decreased significantly. In the sumatriptan, WZYD-M, and WZYD-H groups, the expression of CGRP protein and mRNA in the TNC (*p* < 0.001) was significantly decreased. These results indicate that sumatriptan, WZYD-M, and WZYD-H reversed the levels of CGRP in CM model rats, thereby improving CM symptoms.

In addition, some pharmacodynamic indexes of WZYD in the CM rat model are presented in the Supplementary Materials (Figure S1).

### 2.3. The Potential Mechanism of WZYD in Treating CM Analyzed by Network Pharmacology According to the Components Identified in WZYD

**Figure 2:**
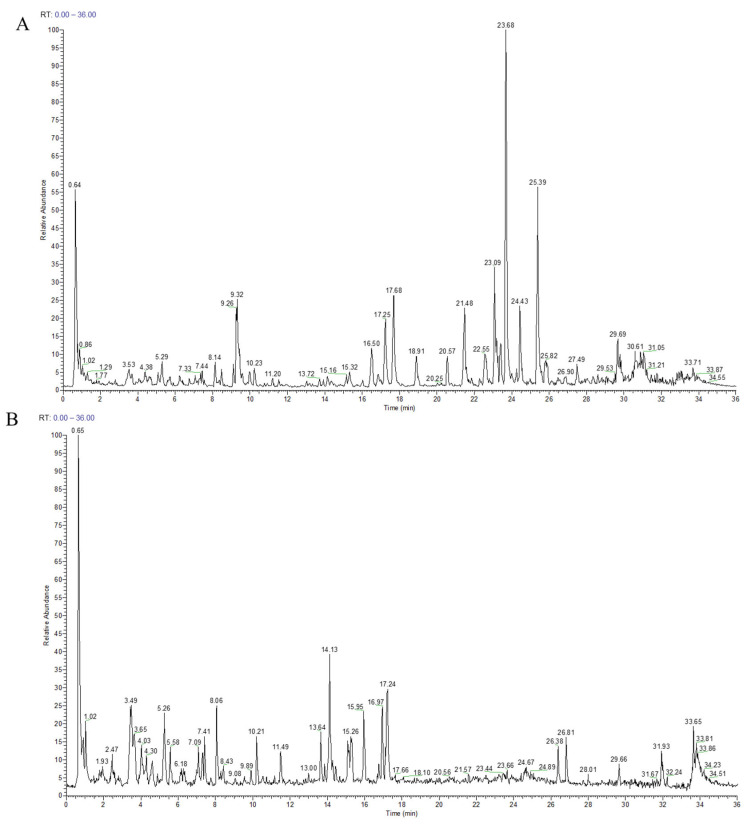
Total ion current diagram of components in WZYD (The green line in the figure is used to mark the retention time of the corresponding chromatographic peak). (**A**) positive ion mode. (**B**) negative ion mode.

**Figure 3:**
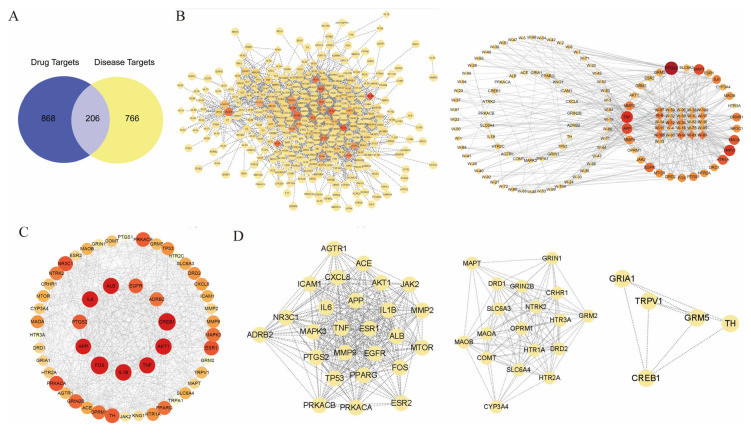
Analytical results of network pharmacology. (**A**) Venn diagram of common target genes between WZYD and CM. (**B**) Key component-target network (diamonds: components; circles: targets). (**C**) The result of PPI analysis of potential targets of WZYD. (**D**) Cluster analysis of key targets in the PPI network.

To explore the therapeutic mechanism of WZYD in CM, we qualitatively analyzed WZYD (Figure 2) and identified 110 components (Table S1). Based on the qualitative analysis results, the potential targets of WZYD were analyzed using network pharmacology. A total of 1074 drug targets and 972 disease targets were identified, and a Venn diagram of the potential active components of WZYD and the therapeutic targets of migraine was drawn (Figure 3A). A protein-protein interaction (PPI) network diagram (Figure 3C) was obtained by importing the potential targets of WZYD in migraine treatment into the String database, which included 205 nodes and 2915 edges. Key targets in the PPI network were visually analyzed using Centiscape 2.2. Based on the criteria of Betweenness ≥ 231.62, Closeness ≥ 0.0023, and Degree ≥ 28.43, 53 key targets were screened. Based on the key targets obtained by PPI screening, the active components were searched in reverse, and a network diagram of the key targets and components of WZYD in the treatment of CM was constructed (Figure 3B). The molecular complex detection (MCODE) algorithm was used to identify densely connected network components and PPI network functional clusters, and three MCODE clusters were generated, which mainly included inflammatory response, 5-HT receptor-related targets, and transient receptor potential (TRP)-related targets (Figure 3D).

**Figure 4:**
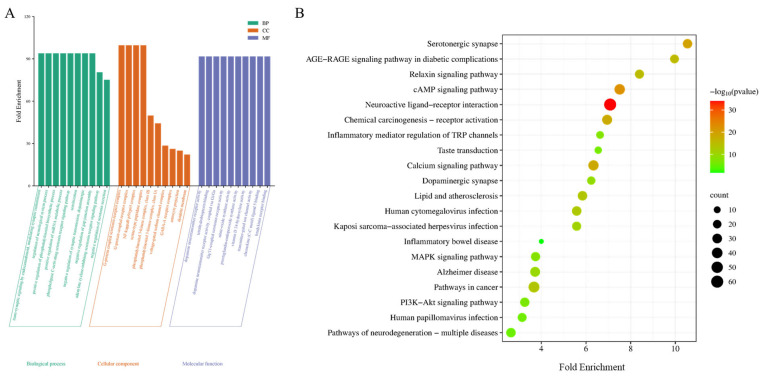
Functional enrichment results of the key targets. (**A**) GO pathway enrichment analysis results. (**B**) Enrichment results for the KEGG signaling pathway.

Using the DAVID database, 206 potential therapeutic targets of WZYD for migraine were analyzed by GO and KEGG enrichment. Histograms and bubble charts were used to visualize the top 10 rich factor results and top 20 paths (Figure 4). The most remarkable enrichment of biological processes mainly includes the regulation of neurological system process, positive regulation of the phosphatidylinositol biosynthetic process, negative regulation of serotonin secretion, and the phospholipase C-activating serotonin receptor signaling pathway. The most remarkable enrichment of cellular component mainly included the G-protein coupled serotonin receptor complex, nuclear factor-*κ*B p50/p65 complex and *γ*-aminobuytric acid type-A (GABA-A) receptor complex. The most significant enrichment of molecular function mainly includes Gq/11-coupled serotonin receptor activity, dopamine neurotransmitter receptor activity, transmitter-gated ion channel activity, and bradykinin receptor binding. The KEGG pathways involved in WZYD’s treatment of CM mainly included the neuroactive ligand-receptor interaction pathway, 5-HT synaptic pathway, cyclic adenosine monophosphate (cAMP) signaling pathway, calcium signaling pathway, inflammatory mediator regulation of TRP channels, phosphatidylinositol-3-kinase/proteinkinase B (PI3K-Akt) signaling pathway, mitogen-activated protein kinases (MAPK) signaling pathway, and dopaminergic synapse, suggesting that WZYD may treat CM mainly by regulating the interaction of neuroactive ligand receptors, 5-HT signaling pathway, and improving inflammatory response. This also provides a basis for further studies on the regulation of 5-HT receptors by WZYD.

### 2.4. Molecular Docking Verification of Key Components with 5-HT1A/3A Receptors

**Figure 5:**
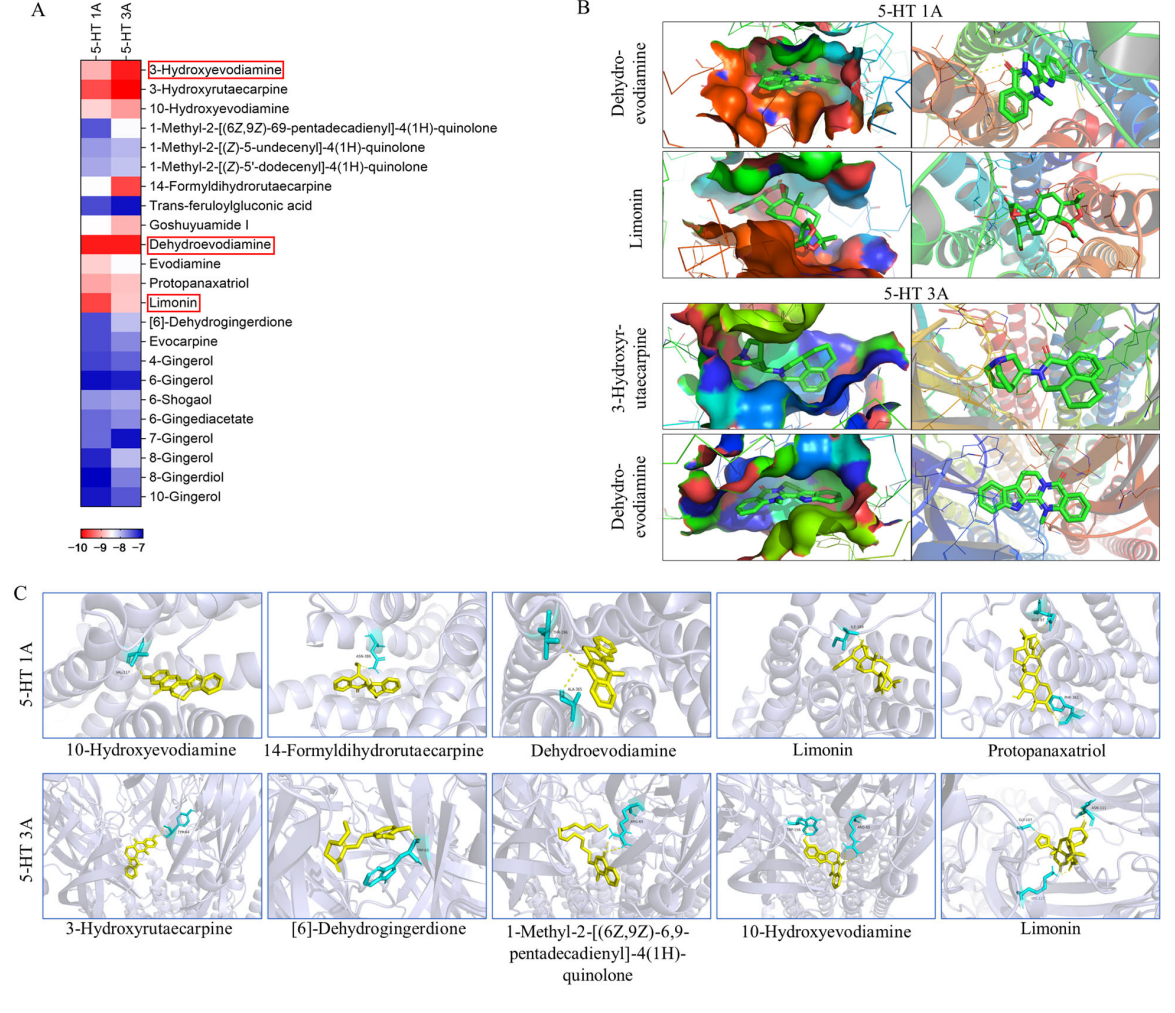
Molecular docking results of key components with 5-HT1A/3A receptors (The red box highlights the two components with the lowest binding energy for 5-HT1A/3A receptors. 5-HT1A: Dehydroevodiamine, Limonin; 5-HT3A: 3-Hydroxyevodiamine, Dehydroevodiamine.). (**A**) Binding energies of the screened key components to the 5-HT1A/3A receptors. (**B**) Docking of the two compounds with the lowest binding energies to the 5-HT1A/3A receptors. (**C**) Components that interact with the amino acid residues of the 5-HT1A/3A receptors (Yellow: compounds; Blue: amino acid residues).

According to the key component-target network screened out by network pharmacology, 23 components with a high correlation with 5-HT1A and 5-HT3A receptors, including 11 alkaloids, nine gingerols, two triterpenoids, and one organic acid (Table S2), were selected. These components were docked with 5-HT1A and 5-HT3A, respectively, forming 46 combinations (Figure 5). Most docked compounds have a strong binding affinity, and each component possesses the capability to enter the active pocket of a protein, enabling them to effectively bind to the active sites of the protein. A binding energy below −5.0 kcal/mol indicates good binding, while a binding energy below −7.0 kcal/mol indicates strong binding activity. In these 46 docking combinations, the minimum binding energy was −10.07 kcal/mol, the maximum binding energy was −6.83 kcal/mol, and the binding energy of 24 combinations was lower than −8 kcal/mol. All of these active compounds showed significant binding affinities for the two receptors. 10-hydroxyevodiamine, 14-formyldihydrorutaecarpine, dehydroevodiamine, limonin, and protopanaxatriol were able to form hydrogen bonds with amino acids such as VAL-117, ASN-386, THR-196, ALA-365, ILE-189, GLN-97, and PHE-361 of the 5-HT1A receptor; 3-hydroxyrutaecarpine, [6]-dehydrogingerdione, 1-methyl-2-[(6Z,9Z)-6,9-pentadecadienyl]-4(1H)-quinolone, 10-hydroxyevodiamine, and 1-methyl-2-[(E,E)-10,30-pentadecadienyl]-4(1H)-quinolone were able to form hydrogen bonds with amino acids such as TYR-64, TRP-63, ARG-65, TRP-156, ASN-111, GLY-107 and LYS-127 of the 5-HT3A receptor. The formation of hydrogen bonds enhanced the interaction between molecules, thereby increasing the stability of the binding between the components and the protein. Components that formed hydrogen bonds may have had higher bioactivity because they could bind more stably to the active sites of the target protein, thus more effectively exerting their pharmacological effects. This also helped the drug components to maintain their activity for a longer period within the body, thereby enhancing the therapeutic effects of the drugs. By analyzing the binding energies between different components and 5-HT1A/3A, it was further confirmed that the components of WZYD have the potential to interact with these two targets.

### 2.5. Effects of WZYD on 5-HT and 5-HT1A/3A Receptors in CM Rats

**Figure 6:**
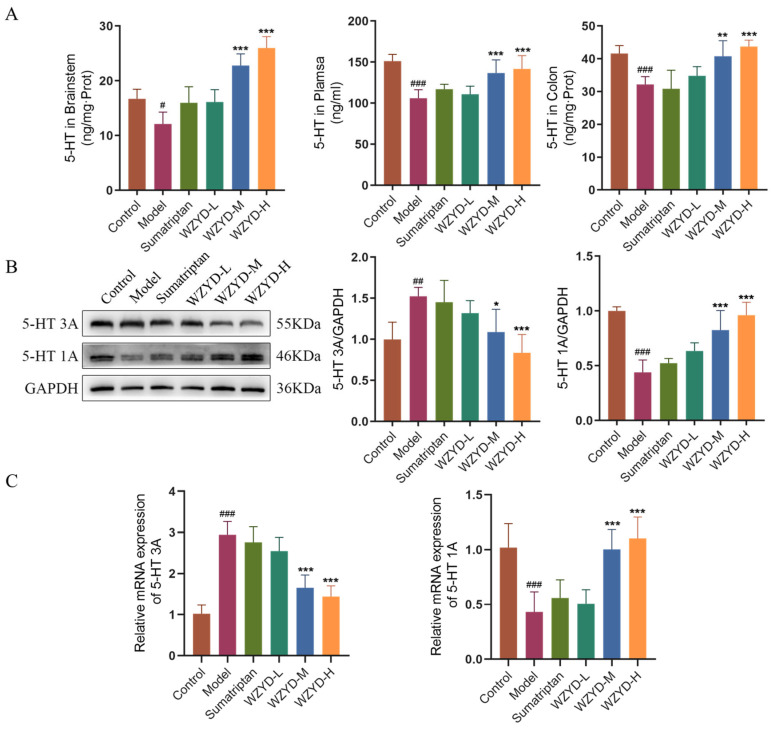
Effects of WZYD on 5-HT and 5-HT1A/3A receptors in CM rats. (**A**) The expression levels of 5-HT in the brainstem, plasma, and colon were measured using ELISA (*n* = 6). (**B**) Relative protein expression of 5-HT1A/3A receptors in TNC (*n* = 5). (**C**) Relative mRNA levels of 5-HT1A/3A receptors in the TNC (*n* = 6). Data are expressed as the mean ± SD. ^#^ *p* < 0.05, ^##^ *p* < 0.01, ^###^ *p* < 0.001 vs. the control group; * *p* < 0.05, ** *p* < 0.01, *** *p* < 0.001 vs. the model group.

5-HT is a key neurotransmitter in the 5-HT pain regulation pathway. The levels of 5-HT in the plasma and TNC, as well as the expression of 5-HT in the colon, were detected (Figure 6A). The results showed that 5-HT levels in the model group were significantly decreased in the plasma (*p* < 0.05), TNC (*p* < 0.001), and colon (*p* < 0.001) compared to those in the control group. Compared with the model group, the 5-HT levels in the WZYD-M and WZYD-H groups increased significantly in the plasma (*p* < 0.001), TNC (*p* < 0.001), and colon (*p* < 0.01). To further verify the regulation of WZYD on 5-HT1A/3A receptors in the brain of CM rats, we compared the protein and mRNA expression of 5-HT1A/3A receptors in the model group to those in the control group and found that the 5-HT3A receptor was significantly increased (*p* < 0.01), while the 5-HT1A receptor was significantly decreased (*p* < 0.001) in the model group. After the intervention of WZYD-M and WZYD-H, the expression of the 5-HT3A receptor protein (*p* < 0.05) and mRNA (*p* < 0.001) was significantly lower than that in the model group, and the expression of the 5-HT1A receptor (*p* < 0.001) was significantly higher than that in the model group. (Figure 6B,C). These results suggest that WZYD may improve CM by regulating the expression of 5-HT1A and 5-HT3A receptors and the 5-HT nerve pathway.

### 2.6. The Regulation of WZYD on 5-HT, 5-HT1A/3A Receptors Were Inhibited by WAY 100635 and SR 57227

**Figure 7:**
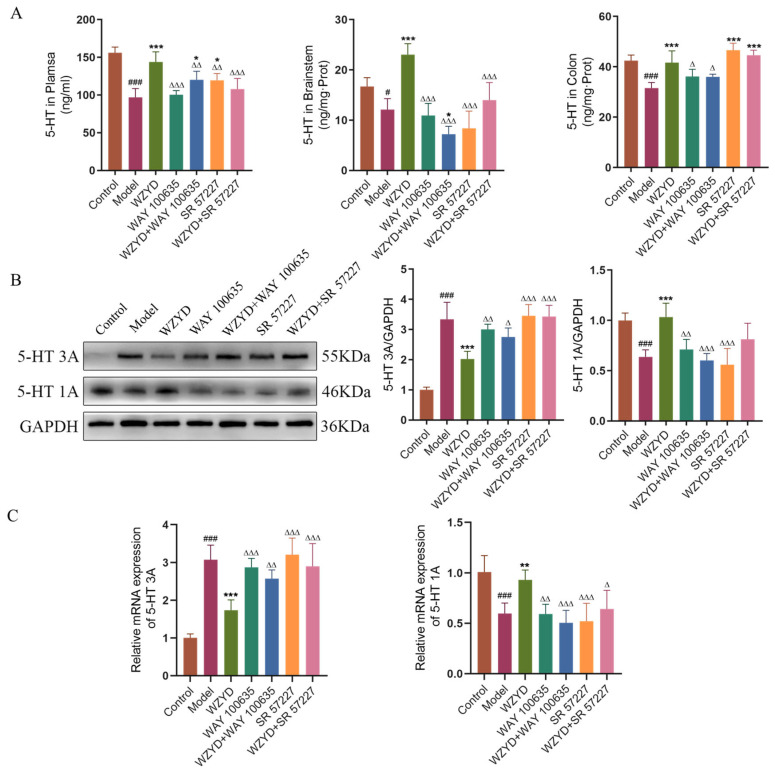
WAY 100635 and SR 57227 inhibited the regulation of WZYD on 5-HT and 5-HT1A/3A receptors in CM rats. (**A**) The expression levels of 5-HT in brainstem, plasma, and colon were measured by ELISA (*n* = 6). (**B**) Relative protein expression of 5-HT1A/3A receptors in TNC (*n* = 5). (**C**) Relative mRNA levels of 5-HT1A/3A receptors in TNC (*n* = 6). Data are expressed as the mean ± SD. *^#^ p* < 0.05, ^###^ *p* < 0.001 vs. the control group; * *p* < 0.05, ** *p* < 0.01, *** *p* < 0.001 vs. the model group; ^∆^ *p* < 0.05, ^∆∆^ *p* < 0.01, ^∆∆∆^ *p* < 0.001 vs. the WZYD group.

The effects of the 5-HT1A receptor antagonist (WAY 100635) and 5-HT3A receptor agonist (SR 57227) on the treatment of 5-HT1A/3A receptor WZYD in CM rats were verified. The dosage of WZYD was selected as the medium dosage with better efficacy and moderate gastric perfusion volume. The changes in 5-HT and 5-HT1A/3A receptors in the brain and colon were detected. The results showed that compared with the control group, 5-HT levels in the plasma, TNC, and colon were significantly decreased (*p* < 0.05). After the intervention of WZYD, the levels of 5-HT were recovered (Figure 7A). After using WAY 100635 and SR 57227, 5-HT levels in the plasma and TNC decreased significantly (*p* < 0.01). In the colon, Only WAY 100635 inhibited the trend of WZYD to increase 5-HT, but this phenomenon was not observed after using SR 57227.

The detection results of the related receptors in the brain stem are shown in Figure 7B,C. Compared with the control group, the protein and mRNA expression of the 5-HT3A receptor in the model group were significantly increased (*p* < 0.001), while the protein and mRNA expression of the 5-HT1A receptor were significantly decreased (*p* < 0.001). After WZYD treatment, the expression of 5-HT1A and 5-HT3A receptors in the model rats was restored. WAY 100635 intervention alone reduced the expression of 5-HT1A receptors, while SR 57227 intervention alone increased the expression of 5-HT3A receptors. These results indicate that WAY 100635 and SR 57227 can be used as receptor antagonists or agonists. When WZYD was combined with WAY 100635 and SR 57227, the restorative effects of WZYD on the 5-HT1A and 5-HT3A receptors were inhibited.

**Figure 8:**
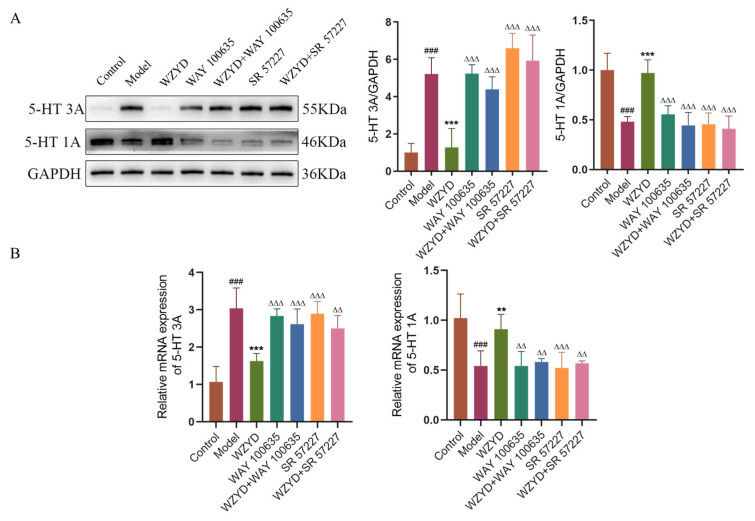
WAY 100635 and SR 57227 inhibited the effect of WZYD on 5-HT1A/3A receptors in the colon of CM rats. (**A**) Relative protein expression of 5-HT1A/3A receptors (*n* = 5). (**B**) Relative mRNA levels of 5-HT1A/3A receptors (*n* = 6). Data are expressed as mean ± SD. ^###^ *p* < 0.001 vs. the control group; ** *p* < 0.01, *** *p* < 0.001 vs. the model group; ^∆∆^ *p* < 0.01, ^∆∆∆^ *p* < 0.001 vs. the WZYD group.

The expression of the related receptors in the colon is shown in Figure 8. Similar to the results in the brainstem, in the model group, the expression of the 5-HT3A receptor increased, while the expression of the 5-HT1A receptor decreased; In WZYD group, the expression of the 5-HT3A receptor decreased and the expression of the 5-HT1A receptor increased, and WAY 100635 and SR 57227 also inhibited the regulation of WZYD on the 5-HT1A/3A receptor. These results indicate that 5-HT1A/3A receptors are involved in the regulation of WZYD in the brain and colon of CM rats. When the expression of 5-HT1A/3A receptors was reversed, the effects of WZYD were inhibited.

### 2.7. The Improvement of Behavioral of CM Rats by WZYD Was Inhibited When WZYD Was Combined with WAY 100635 and SR 57227

**Figure 9:**
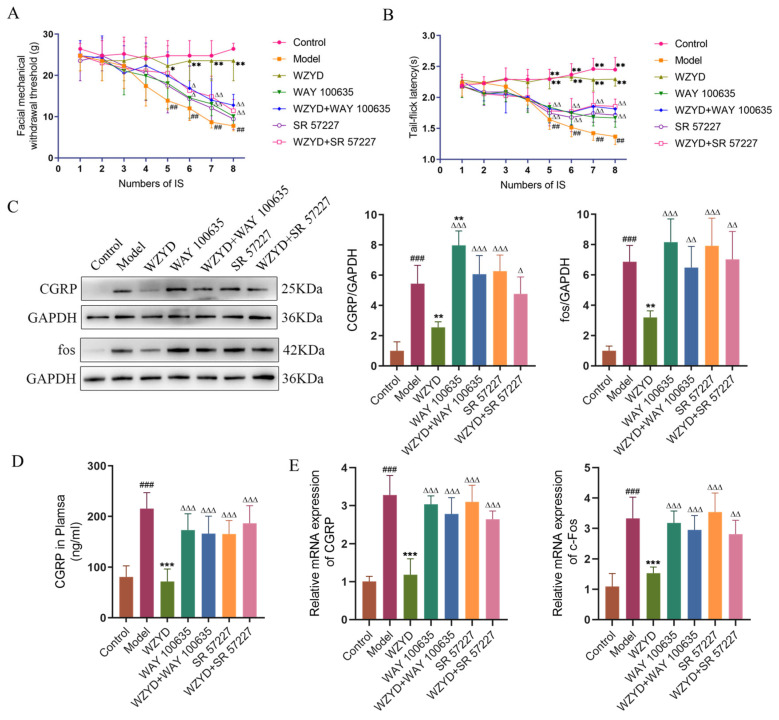
WAY 100635 and SR 57227 inhibited the effects of WZYD in CM rats. (**A**) Facial mechanical withdrawal threshold (*n* = 9). (**B**) Tail-flick latency (*n* = 9). (**C**) Relative protein expression of CGRP and fos in the TNC (*n* = 5). (**D**) The expression level of CGRP in TNC was measured using ELISA (*n* = 6). (**E**) Relative mRNA levels of CGRP and c-Fos in the TNC (*n* = 6). Data are expressed as the mean ± SD. ^##^ *p* < 0.01, ^###^ *p* < 0.001 vs. the control group; * *p* < 0.05, ** *p* < 0.01, *** *p* < 0.001 vs. the model group; ^∆^ *p* < 0.05, ^∆∆^ *p* < 0.01, ^∆∆∆^ *p* < 0.001 vs. the WZYD group.

To reveal the pivotal role of the 5-HT1A/3A receptor in the treatment of CM by WZYD, we detected the changes in pain threshold in CM rats after intervention with WAY 100635 and SR 57227. Compared with the control group, the thresholds of orbital mechanical pain and tail-flick latency in the model group were significantly reduced after the fourth IS stimulation, while the two pain thresholds in the WZYD group were not significantly reduced. However, when we used WAY 100635 and SR 57227, the effect of WZYD on increasing the pain threshold was inhibited (Figure 9A,B). These results suggest that WAY 100635 and SR 57227 could blunt WZYD’s upregulation of pain threshold.

### 2.8. The Improvement of WZYD on Neuron Activation and Neuroinflammation in CM Rats Was Inhibited When WZYD Was Combined with WAY 100635 and SR 57227

**Figure 10:**
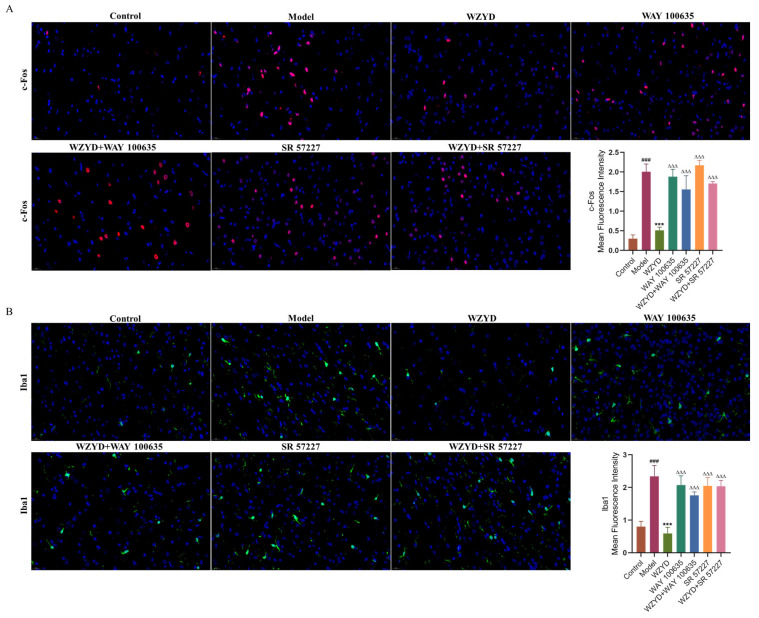
WAY 100635 and SR 57227 inhibited the regulatory effect of WZYD on c-Fos and Iba1 in the brainstem of CM rats. (**A**) Immunofluorescence staining of c-Fos (red) and DAPI (blue). (**B**) Immunofluorescence staining of Iba1 (green) and DAPI (blue). Scale bar = 20 μm. Data are expressed as the mean ± SD. (*n* = 3) ^###^ *p* < 0.001 vs. the control group; *** *p* < 0.001 vs. the model group; ^∆∆∆^ *p* < 0.001 vs. the WZYD group.

By measuring pharmacodynamic indicators related to the pathogenesis of migraine, the effects of WAY 100635 and SR 57227 on the efficacy of WZYD were further evaluated. The results showed that CGRP and c-Fos levels in the model group were significantly higher (*p* < 0.001) than those in the control group. After the intervention of WZYD, the expression of CGRP and c-Fos decreased (Figure 9C–E). However, the levels of CGRP and c-Fos increased significantly (*p* < 0.05) when WZYD was combined with WAY 100635 and SR 57227. Compared with the control group, the fluorescence intensities of c-Fos and ionized calcium-binding adapter molecule 1 (Iba1) in the model group increased, but the fluorescence intensities of c-Fos and Iba1 decreased after WZYD administration. Compared with the WZYD group, the fluorescence intensities of c-Fos and Iba1 increased significantly after treatment with WAY 100635/SR 57227 in combination with WZYD (Figure 10), and the effects of WZYD in reducing c-Fos and Iba1 were also inhibited to some extent. These data indicate that the 5-HT1A/3A receptor was involved in the treatment of CM by WZYD in a rat model of CM.

### 2.9. WZYD Improves CM by Regulating 5-HT1A and 3A Receptors-Mediated CREB Signaling Pathway

**Figure 11:**
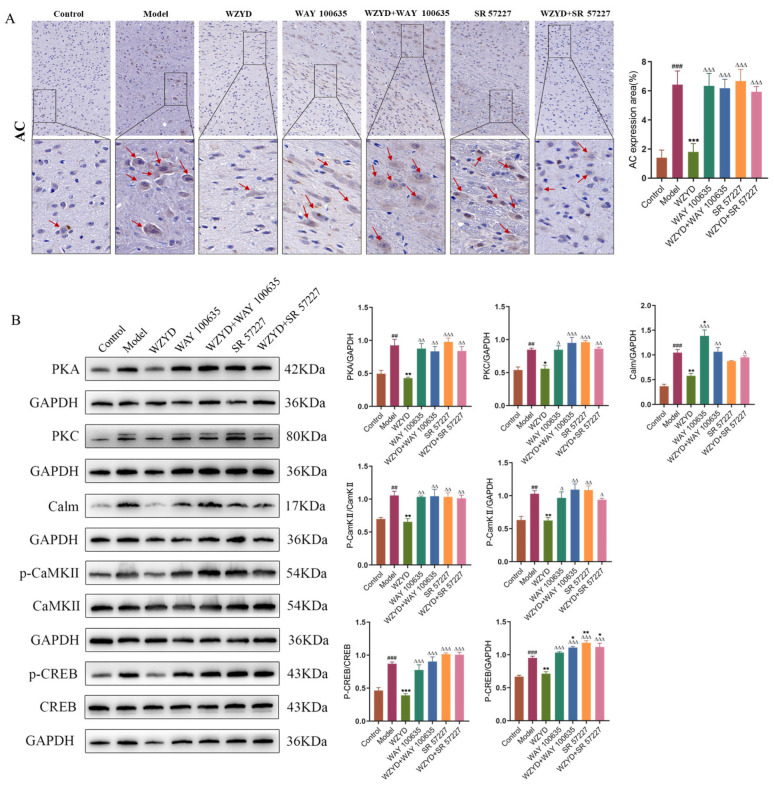
Effect of WZYD on 5-HT1A and 3A receptor-mediated CREB signaling pathway protein in the brainstem (The red arrows indicate positive signals). (**A**) Immunohistochemical images of the AC in the brainstem. Scale bar = 20 μm. (**B**) Relative protein expression of AC, PKA, PKC, Calm, p-CaMKII/CaMKII, and p-CREB/CREB in TNC (*n* = 3). Data are expressed as the mean ± SD. ^##^ *p* < 0.01, ^###^ *p* < 0.001 vs. the control group; * *p* < 0.05, ** *p* < 0.01, *** *p* < 0.001 vs. the model group; ^∆^ *p* < 0.05, ^∆∆^ *p* < 0.01, ^∆∆∆^ *p* < 0.001 vs. the WZYD group.

After we identified the critical role of the 5-HT1A/3A receptor, we investigated the potential molecular mechanisms of WZYD in CM therapy (Figure 11). The expression of adenyl cyclase (AC), protein kinase A (PKA), protein kinase C (PKC), calmodulin (Calm), phosphorylated calcium/calmodulin-dependent protein kinase II (p-CaMKII), calcium/calmodulin-dependent protein kinase II (CaMKII), phosphorylated cAMP-response element binding protein (p-CREB), and cAMP-response element binding protein (CREB) in the brains of CM rats was significantly increased after stimulation with IS (*p* < 0.01). After WZYD treatment, the expression levels of AC, PKA, PKC, Calm, p-CaMKII/CaMKII, and p-CREB/CREB were significantly decreased (*p* < 0.05). Compared with the WZYD group, the expression of AC, PKA, PKC, Calm, p-CaMKII/CaMKII, and p-CREB/CREB was significantly increased after intervention with WAY 100635 and SR 57227 (*p* < 0.05). These results indicate that WZYD regulates the 5-HT1A and 3A receptor-mediated CREB signaling pathway by regulating 5-HT1A/3A receptors, inhibiting pain signal conduction, and improving CM.

**Figure 12:**
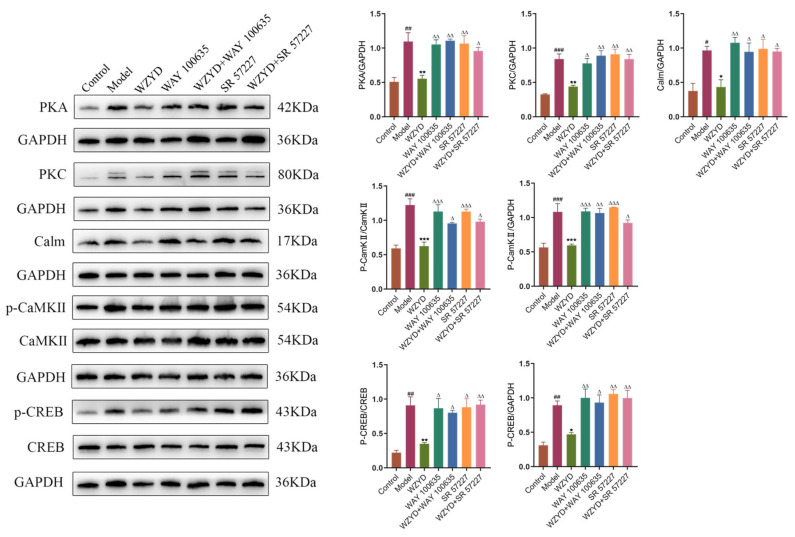
Effect of WZYD on 5-HT1A and 3A receptor-mediated CREB signaling pathway proteins in the colon. Relative protein expression of PKA, PKC, Calm, p-CaMKII/CaMKII, and p-CREB/CREB in the colon (*n* = 3). Data are expressed as the mean ± SD. ^#^ *p* < 0.05, ^##^ *p* < 0.01, ^###^ *p* < 0.001 vs. the control group; * *p* < 0.05, ** *p* < 0.01, *** *p* < 0.001 vs. the model group; ^∆^ *p* < 0.05, ^∆∆^ *p* < 0.01, ^∆∆∆^ *p* < 0.001 vs. the WZYD group (*n* = 3).

WZYD regulation of PKA, PKC, Calm, p-CaMKII/CaMKII, and p-CREB/CREB in the colon showed results similar to those in the brain. Compared with the control group, the expression levels of PKA, PKC, Calm, p-CaMKII/CaMKII, and p-CREB/CREB in the model group were significantly increased, while the expression of these proteins decreased after WZYD treatment. Moreover, the regulatory effects of WZYD on PKA, PKC, Calm, p-CaMKII/CaMKII, and p-CREB/CREB were inhibited by WAY 100635 and SR 57227 (Figure 12).

## 3. Discussion

Our results suggest that the therapeutic effect of WZYD on CM may be associated with 5-HT and 5-HT1A/3A receptors, as it reversed the decreased expression of 5-HT1A receptors and the increased expression of 5-HT3A receptors in the brain and colon of CM rats. When we used a 5-HT1A receptor antagonist (WAY 100635) and 5-HT3A receptor agonist (SR 57227), the effects of WZYD in increasing 5-HT levels in the brain and decreasing CGRP, c-Fos, and Iba1 were inhibited, and the regulatory actions of WZYD on the 5-HT1A/3A receptors in both the brain and colon were also reversed. The 5-HT1A receptor antagonist (WAY 100635) and 5-HT3A receptor agonist (SR 57227) also inhibited the regulatory effects of WZYD on PKA, PKC, Calm, p-CaMKII/CaMKII, and p-CREB/CREB. From these results, we can confirm that the treatment of CM with WZYD was closely related to the regulation of 5-HT1A/3A receptors and their downstream signaling pathways. WZYD has a bidirectional regulatory effect, simultaneously increasing the expression of the 5-HT1A receptor and decreasing the expression of the 5-HT3A receptor in both the brain and colon. This therapeutic feature has great advantages; WZYD not only effectively alleviates headache symptoms but also improves related gastrointestinal symptoms, exerting a brain-gut co-treatment effect for CM.

Additionally, we found that the therapeutic effect of low-dose WZYD on CM was not satisfactory (including the impact on mechanical pain threshold, thermal pain threshold, 5-HT, and CGRP), while both medium and high doses of WZYD showed significant therapeutic effects. This indicates that WZYD needs to reach a certain concentration in the body to effectively act on the target, and an insufficient dosage is difficult to exert the expected therapeutic effect.

Migraine and gastrointestinal diseases are interrelated, and suffering from one disease increases the risk of developing the other disease [22]. Migraines and gastrointestinal diseases are both associated with the dysfunction of 5-HT receptors. Changes in the level of 5-HT in both the central and peripheral regions of migraine patients affect the corresponding receptors, which also leads to functional disorders in the gastrointestinal receptors, thereby increasing the risk of gastrointestinal diseases. The level of 5-HT in the gastrointestinal tract of patients with gastrointestinal diseases changes, thereby stimulating the sensory neurons located in the gastrointestinal tract. After vagal and spinal neurons located in the intestines are activated, they activate the central nervous system. Frequent activation of the central nervous system leads to central sensitization of pain in CM [23,24]. Therefore, the treatment of CM should not only focus on blocking the central pain pathway, but also on intervening in gastrointestinal symptoms. By adjusting gastrointestinal function, it is feasible to regulate brain-gut axis function and achieve the therapeutic goals. It is recorded in “Shang Han Lun”: “Retching, vomiting saliva, and headache are primarily treated with WZYD’’. This also illustrates that WZYD not only treats CM but also improves gastrointestinal diseases, alleviates symptoms such as retching and drooling foaming at the mouth in CM patients, and functions simultaneously in both the central and peripheral nervous systems.

Currently, various types of drugs, including CGRP receptor antagonists, nonsteroidal anti-inflammatory drugs (NSAIDs), and triptans, have been developed for the treatment of CM. However, these medications often have significant side effects. For example, NSAIDs cause gastrointestinal discomfort, such as abdominal pain, nausea, vomiting, and, heartburn. Some patients using CGRP receptor antagonists may experience gastrointestinal discomfort, including nausea and vomiting. Triptans, which have vasoconstrictive effects, are contraindicated in patients with a history of cardiovascular disease, and their long-term use leads to medication-overuse headache. Given that more than 70% of patients with CM experience nausea, antiemetics are often used in combination with other drugs to treat migraines [4,5]. However, both the side effects of the drugs and the use of multiple medications add to the burden on the patients. Triptans and ditans target the 5-HT1B/1D/1F receptors, which are primarily distributed in the central nervous system, smooth muscle, and endothelial cells of cranial blood vessels, with limited distribution in the gastrointestinal tract [15,25,26]. Although these drugs improve CM by modulating the release of central neurotransmitters, they do not improve gastrointestinal symptoms or block the interaction between the brain and intestine. This limitation may contribute to the recurrent nature of headaches. Sumatriptan is an approved and widely used drug in clinical practice for the treatment of migraines and has established efficacy and safety. It is a highly selective 5-HT1B/1D receptor agonist, similar to the effect of WZYD we found. When administered orally, it has shown good therapeutic effects in treating migraines [27]. We used a clinically equivalent dose and avoided continuous administration to prevent medication-overuse headaches. By comparing the effects of WZYD and sumatriptan on indicators such as 5-HT and CGRP, we can gain a deeper understanding of the therapeutic pathways of WZYD in CM and the differences in their mechanisms of action. This is the reason we chose sumatriptan as the positive control drug. Our results show that sumatriptan does not significantly regulate 5-HT levels, but it reduces the expression of CGRP, a vasodilatory neuropeptide, and contracts intracranial blood vessels, thereby alleviating migraines. Additionally, sumatriptan has no effect on 5-HT1A/3A receptors, which is consistent with its therapeutic characteristics. However, since the 5-HT1B/1D receptors have limited effects in the gut, this may also be one of the reasons long-term use of sumatriptan leads to significant side effects. This also illustrates the significant shortcomings of treating CM solely via the central nervous system. Such an approach cannot comprehensively improve symptoms and may even increase treatment side effects. Therefore, there should be a greater emphasis on the holistic regulation of the brain-gut axis to enhance the therapeutic efficacy.

The 5-HT1A receptor is an inhibitory G-protein-coupled receptor that is widely distributed in the central nervous system and the enteric nervous system. It plays an important role in regulating neurotransmitter release, mood, cognitive function, and the development of gastrointestinal diseases. The 5-HT1A receptor is primarily located on the presynaptic membrane, where it acts as an autoreceptor. Its activation can inhibit neuronal firing activity and reduce the synthesis and release of 5-HT. This negative feedback mechanism helps regulate the levels of 5-HT in the synaptic cleft and maintain the balance of neurotransmitters [17,18]. In this study, we found that the levels of 5-HT and 5-HT1A receptors were reduced in the brains and colons of CM rats. This disruption of the balance leads to abnormal neuronal excitation, making the brain and colon more sensitive to noxious stimuli.

The 5-HT3A receptor is a ligand-gated ion channel receptor that primarily influences neuronal signaling by regulating the opening of ion channels. It is widely distributed in the central nervous system, where its activation can lead to rapid neuronal depolarization and mediate a fast excitatory neurotransmission. This activation can also enhance the transmission of pain signals. In the peripheral nervous system, the 5-HT3A receptor is mainly found in the myenteric plexus and submucosal plexus of the gastrointestinal tract, where it is involved in regulating gastrointestinal motility and secretory functions [19,20]. The 5-HT3A receptor is a key target for chemotherapy-induced nausea and vomiting. By blocking the 5-HT3A receptor, the excitability of the vomiting center is reduced, effectively alleviating nausea and vomiting caused by chemotherapy.

**Figure 13:**
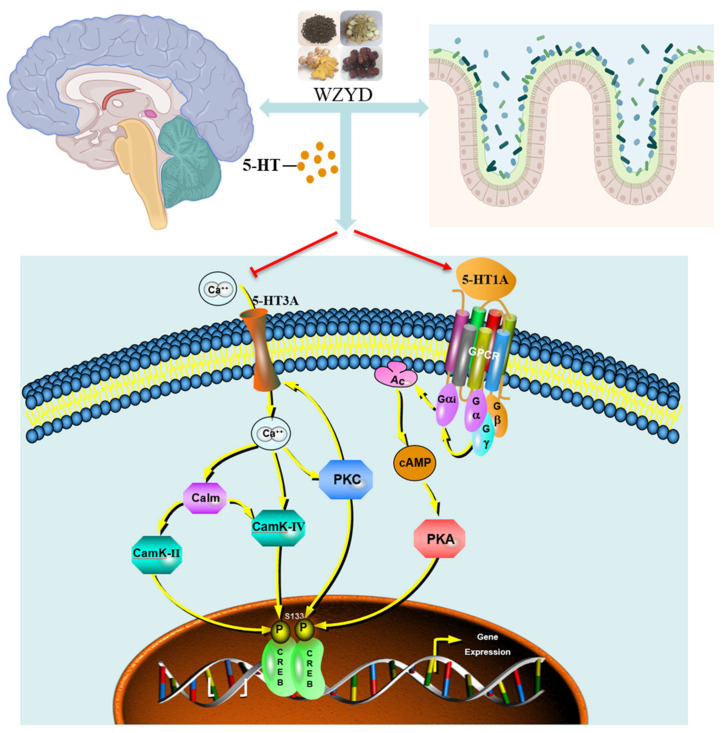
WZYD alleviates CM via the 5-HT1A and 3A receptor-mediated CREB signaling pathway.

From our research results, we find that WZYD not only increased the levels of 5-HT in the brain and colon but also specifically increased the expression of 5-HT1A receptors and decreased the expression of 5-HT3A receptors. This indicates that WZYD alleviates CM by simultaneously regulating the content of 5-HT and the excitation/inhibition of the 5-HT1A/3A receptor at the same time, thereby regulating the functions of the brain and colon. In the CM rat brain, the decreased expression of 5-HT1A receptors increases the sensitivity of sensory nerve fibers to pain stimuli and promotes the triggering of the vomiting reflex. The increased expression of 5-HT3A receptors enhances neuronal excitability, promotes the transmission of pain signals, thereby exacerbating pain, and increases the excitability of the vomiting center, promoting the generation of vomiting signals, thus inducing vomiting. In the colon of CM rats, the decreased expression of 5-HT1A receptors and the increased expression of 5-HT3A receptors similarly increase the sensitivity of sensory nerve fibers to pain stimuli and that of the gastrointestinal tract to emetic stimuli. Importantly, dysfunction of 5-HT1A/3A receptors on the sensory nerve fibers of the gastrointestinal tract also leads to increased central pain sensitivity [28,29,30,31]. Due to the mutual interference between the brain and intestine, only one aspect of treatment may lead to recurrent symptoms. In communication of the brain-gut axis, 5-HT1A/3A receptors can act through the vagus nerve and humoral pathways. After 5-HT is released, it interacts with receptors in the enteric nervous system, regulating intestinal motility and inducing further signal conduction along the vagus nerve. The afferent signals of the vagus nerve then propagate further to the central nervous system, regulating its neural activity [32]. In addition, 5-HT1A/3A receptors regulate the levels of 5-HT in the blood [17,33]. Through blood circulation, 5-HT affects brain areas outside the blood-brain barrier and transmits signals to the brainstem [34]. Therefore, the treatment of CM requires attention to the regulation of receptors in both the brain and the intestine. In this study, we found that 5-HT1A/3A receptors may be important targets for WZYD in the regulation of brain and intestinal functions. WZYD enhanced the expression of 5-HT and 5-HT1A receptors in the brain and colon, while decreasing the expression of the 5-HT3A receptor. This modulation subsequently inhibited the expression of PKA, PKC, Calm, p-CaMKII/CaMKII, and p-CREB/CREB, thereby improving CM and alleviating gastrointestinal symptoms (Figure 13).

CREB is a key transcription factor that plays a crucial role in synaptic function, neuroplasticity, gastrointestinal function, and pain perception by regulating gene expression [35]. Studies have found that the activation of the 5-HT1A receptor reduces the levels of PKA and p-CREB, thereby decreasing neuronal excitability and inhibiting pain sensitivity [36]. Blocking the 5-HT1A receptor can increase CaMKII and CREB levels, thus inhibiting the analgesic effect of acupoint catgut embedding (ACE) [37]. Activating 5-HT3 receptors can elevate the level of CREB through the CaMKII-CREB signaling pathway, leading to the occurrence of postoperative cognitive dysfunction [38]. In CM, the activation of CREB is associated with multiple proteins, including PKA, PKC, Calm, and CaMKII [39,40,41]. WZYD regulated the expression of PKA, PKC, Calm, p-CaMKII/CaMKII, and p-CREB/CREB in the brain and colon. However, when 5-HT1A receptor antagonists and 5-HT3A receptor agonists are used for intervention, the regulation of these proteins by WZYD is inhibited. This indicates that WZYD regulates the expression of downstream proteins and inhibits the phosphorylation of CREB by modulating 5-HT1A/3A receptors, thereby exerting a therapeutic effect on CM.

In a study, researchers found that in a mouse model of migraine induced by nitroglycerin, there was a significant increase in inflammatory factors such as TNF-*α* and IL-1*β* in the colon and a decrease in the integrity of the colonic epithelial barrier. By regulating the balance of Nrf2/NOX2 in the brain and colon, nerve and related intestinal damage during migraines can be reduced [22]. Impaired intestinal function allows bacterial metabolites and inflammatory factors to enter the bloodstream. These inflammatory factors stimulate trigeminal nerve endings, releasing CGRP and causing blood vessel dilation and migraines [34]. Gastrointestinal sensory information can be transmitted to the brain via the vagus nerve or the enteric nervous system. Abnormalities in 5-HT and 5-HT receptors can activate the vagus nerve and trigger migraines [42]. These research findings demonstrate the interaction between the brain and the gut in the pathogenesis of migraine. Our experimental results showed that the 5-HT1A/3A receptors in the brain and colon of CM rats changed. After administration of WZYD, the expression of 5-HT1A/3A receptors in the brain and colon was restored, which is consistent with its therapeutic effects on CM and its ability to improve gastrointestinal symptoms. As gut dysfunction is closely related to migraines, we believe that the treatment of migraines should also focus on the regulation of gut function. This is consistent with the mechanism of action of WZYD in treating migraine in traditional Chinese medicine.

Prior to this, we had already conducted some research on the mechanism by which WZYD treats CM through the brain-gut axis. First, we found that WZYD could increase the levels of 5-HT in the brain and colon, and the mechanisms of increasing 5-HT in the brain and colon were not exactly the same. In the brain, it did not increase the synthesis of 5-HT but instead inhibited the metabolism of 5-HT and increased the content of 5-HT in the synaptic cleft. In the colon, it increased the synthesis of 5-HT and enhanced the transport of 5-HT into the bloodstream, preventing excessive 5-HT levels from causing intestinal dysfunction [8]. Additionally, WZYD improved the gut microbiota imbalance in CM rats induced by IS. Subsequently, the role of gut microbiota in WZYD’s treatment of CM was confirmed through antibiotic interference. It also revealed the therapeutic effect of WZYD on CM rats with anxiety-depressive-like behaviors mediated by the gut microbiota [43]; A recent study has shown that there is a close link between diet and migraines. Some foods (such as chocolate, caffeine, and alcohol) may trigger migraine attacks, while dietary approaches, such as a low-fat diet and ketogenic diet, may help prevent migraines. This also shows that regulating intestinal flora is beneficial for the treatment of migraine [42,44]. In this study, we targeted the 5-HT1A/3A receptors, which function in both the brain and gut, and found that the therapeutic advantages of WZYD differ from those of other drugs. By simultaneously regulating the function of 5-HT1A/3A receptors in the brain and colon and then regulating the CREB signaling pathway they mediate, WZYD treats CM while also improving intestinal function and avoiding recurrent headaches caused by intestinal dysfunction. In summary, through these studies, we have clarified the mechanism of action of WZYD in treating CM from multiple aspects, including 5-HT, 5-HT receptors, and gut microbiota. This is beneficial for avoiding the drug resistance problem easily caused by single-target drugs [45,46]. It is helpful to deeply understand the pathological process of CM and provide a basis for further research.

We are fully aware of the crucial role of human studies in validating the therapeutic potential of WZYD. Although we have not yet conducted human studies on WZYD. However, existing clinical studies have shown that WZYD has good therapeutic effects on migraines and some gastrointestinal diseases (chronic non-atrophic gastritis and vomiting) [7,47,48]. Although current clinical studies have not directly explored the effect of WZYD on 5-HT receptors, in clinical practice, 5-HT1B/1D1F receptor agonists have been widely used in the treatment of migraines [49]; 5-HT3 receptor antagonists are used for chemotherapy-induced nausea and vomiting [50]; and 5-HT3, 5-HT4, and 5-HT7 receptors are involved in the prevention and treatment of postoperative gastrointestinal dysfunction in patients undergoing thoracoscopy [51]. The therapeutic effects of WZYD on migraine and gastrointestinal diseases are similar to those of 5-HT receptor agonists or antagonists. These studies provide a basis for the clinical use of WZYD in treating CM and related gastrointestinal symptoms through the regulation of 5-HT receptors. In the future, further verification of the mechanism of action of WZYD on 5-HT receptors in human studies can clarify its clinical application.

In conclusion, our results indicate that WZYD regulates 5-HT and 5-HT1A/3A receptors in both the brain and colon, thereby modulating the 5-HT1A and 3A receptor-mediated CREB signaling pathways. While treating CM, WZYD also improved gastrointestinal symptoms, exerting a brain-gut co-treatment effect for CM. This is an advantage that many drugs do not possess. Many drugs that solely target 5-HT or 5-HT receptors often have severe side effects and lead to drug tolerance or overuse headache with long-term use. In existing research on the mechanisms of TCM for treating CM, few studies have focused on the regulation of the 5-HT receptor and its downstream signaling pathway in the brain and intestine at the same time. The results of this study expand our understanding of the mechanisms and advantages of WZYD in treating CM.

This study has some limitations. Due to the limitations of the rat model, the responses of nausea and vomiting could not be observed; therefore, we substituted them with the detection of neurotransmitters. Additionally, although some studies have mentioned the interaction between the central and peripheral systems in CM, there is still a lack of direct evidence, which requires further research. Considering that the physiological factors and hormone levels of female rats may have an impact on the mechanism of WZYD in treating CM, this study chose male rats, lacking the research of female rats. Although the conclusions drawn from animal experiments are meaningful, further verification is needed in human studies. The results of future human trials will be crucial in determining the actual therapeutic value of WZYD.

## 4. Materials and Methods

### 4.1. Drugs and Reagents

WZYD was prepared from four kind of decoction piece, including Euodiae Fructus (nearly ripe fruit of *Euodia rutaecarpa* (Juss.) Benth.), Ginseng Radix et Rhizoma (root or rhizome of *Panax ginseng* C. A. Mey.), Jujubae Fructus (ripe fruit of *Ziziphus jujuba* Mill.), and Zingiberis Rhizoma Recens (rhizome of *Zingiber officinale* Rosc.), which were purchased from Anguo Chinese Medicine Market in Hebei, China. The proportions of the four herbs were Euodiae Fructus (69 g), Ginseng Radix et Rhizoma (41.4 g), Zingiberis Rhizoma Recens (82.8 g), and Jujubae Fructus (36 g). The mixture was soaked in 1400 mL distilled water for 30 min and then decocted until 400 mL remained. The extractive solution was concentrated and dried in a vacuum dry box to obtain a dry powder.

IS contained 2 mmol/L histamine (>97%) and 2 mmol/L serotonin hydrochloride (≥98%) purchased from Sigma–Aldrich Inc. (Saint Louis, MO, USA), and 0.2 mmol/L prostaglandin (≥98%) purchased from Yeasen Biotechnology Co., Ltd. (Shanghai, China) and 2 mmol/L bradykinin (≥98%) purchased from Shanghai Aladdin Biochemical Technology Co., Ltd. (Shanghai, China), which were all dissolved in HEPES (≥99%, Beijing Solarbio Science & Technology Co., Ltd., Beijing, China). The positive control drug, sumatriptan succinate, was purchased from Shanghai Macklin Biochemical Technology Co., Ltd. (Shanghai, China). The 5-HT1A receptor antagonist (WAY 100635) and 5-HT3A receptor agonist (SR 57227) were purchased from Shanghai Bide Pharmaceutical Technology Co., Ltd. (Shanghai, China).

### 4.2. Animal Experimental Design

Male Sprague-Dawley rats weighing 200 ± 20 g were purchased from Beijing Vital River Laboratory Animal Technology Co., Ltd. Rats were kept in a specific pathogen-free (SPF) experimental animal center of Capital Medical University (12 h light/dark cycle and 22–24 °C) and provided with ad libitum access to standard chow and clean water. All experiments were approved by the Animal Ethics Committee of Capital Medical University (Authorization number: AEEI-2020-002).

The surgical procedure was as follows: using a small-animal gas anesthesia machine (4% isoflurane for induction and 1.5% for maintenance), the rats were anesthetized. Then, the scalp on the top of the head was cut open to expose the anterior fontanelle. On the left frontal bone, 1.5 mm to bregma and 1.5 mm to the superior sagittal sinus, a hole with a diameter of about 1 mm was drilled, and the dura mater was exposed for the placement of the drug-administration cannula. At the same time, three small holes were drilled near the hole in a triangular shape for the placement of the fixing screws. The cannula and three screws were fixed using dental cement. After the cement dried, the skin was sutured to ensure that the cannula was outside the skin, and penicillin (1 million IU/kg) was injected to prevent infection.

In this study, two animal experiments were conducted. In the first experiment, fifty-four rats were randomly divided into six groups (*n* = 9): Control, model, sumatriptan (5.83 mg/kg^3^ d), low-dose (1.78 g/kg∙d), medium-dose (3.56 g/kg∙d), and high-dose (7.12 g/kg∙d) WZYD groups. HEPES buffer (pH 7.4) or IS (10 μL) was infused into the dura mater of the control or other groups through a cannula. The stimulation was performed once every three days, for a total of eight times. The rats in the sumatriptan group were intragastrically administered sumatriptan succinate while IS was applied, and the same volume of distilled water was administered at other times. The control and model groups were administered distilled water, and the WZYD group was administered different doses of WZYD. After 22 days, the rats were anesthetized, and jugular vein plasma was collected. Subsequently, the proximal part of the colon (5 cm) was removed, thoroughly rinsed with saline solution, and rapidly frozen in liquid nitrogen. The brain tissues were then removed. The TNC was separated from the junction between the cerebellum and the medulla oblongata in the posterior part of the rat’s brain (approximately 14.0 to 14.5 mm from the back of the anterior fontanelle, 1.5 to 2.0 mm from the sides of the midline, and 7.0 to 8.0 mm below the surface of the brain, according to “Paxinos and Watson, The Rat Brain in Stereotaxic Coordinates”), and rapidly frozen in liquid nitrogen. All samples were stored in a refrigerator at −80 °C.

**Figure 14:**
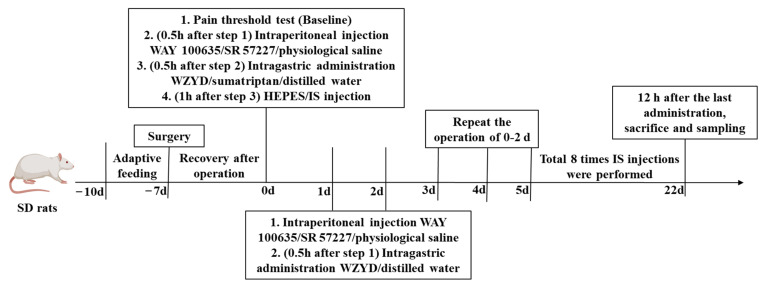
Experimental procedure.

In the second experiment, sixty-three animals were divided into control, model, WZYD, WAY 100635, WZYD + WAY 100635, SR 57227, and WZYD + SR 57227 groups. A medium dose of WZYD (3.56 g/kg∙d) was administered individually or simultaneously with a 5-HT1A receptor antagonist (WAY 100635, 1 mg/kg∙d, i.p.) or a 5-HT3A receptor agonist (SR 57227, 1 mg/kg∙d, i.p.) every day. The WAY 100635 and SR 57227 groups were administered an equal volume of vehicle. After the experiment, jugular vein plasma, brain, and colon tissues were collected, and the proximal colon and TNC were separated according to the above method and stored in the refrigerator at −80 °C. The experimental steps are shown in Figure 14.

### 4.3. Behavioral Test

Von Frey filament (Aesthesio, Gemonio, VA, Italy) was used to test the threshold of facial mechanical withdrawal in rats before the HEPES or IS injection. Before the test commenced, the rats were allowed a 30-min adaptation period within the testing apparatus. A filament was gently applied to the rat’s periorbital area, exerting sufficient pressure to bend the filament and maintain contact for 1–2 s. If no positive response was observed (such as the rat withdrawing, dodging, or scratching), a filament of a higher grade was selected; if a positive response was observed, a filament of a lower grade was selected. By continuously adjusting the specifications of the Von Frey filaments with different hardness levels, the process continued until the stimulus elicited a positive behavior. The gram value at this point was recorded as the rat’s threshold of facial mechanical. Each test was repeated three times, with at least a 2-min interval between each test.

Detection of thermal pain threshold in rats by tail-flick latency (YLS-12A, Shandong, China). Before the test began, the rat was placed into the apparatus to adapt to the equipment for 30 min. The rat’s tail was gently positioned in the equipment’s sensing area, and heat radiation was applied 4 cm from the end of the rat’s tail. When the rat exhibited a tail-flick response, the time of the rat’s tail movement reaction was recorded. This measurement was repeated three times, with a 10-min interval between each test, and the average of the three measurements was taken as the rat’s thermal pain threshold.

### 4.4. ELISA Analysis

To measure the levels of 5-HT and CGRP in the brain stem and colon tissues, the tissues were homogenized in PBS on ice, centrifuged at 3000 r/min and 4 °C for 20 min, and the supernatant was collected for detection. The levels of CGRP in plasma and 5-HT in the plasma, brain stem, and colon were detected using rat CGRP and 5-HT ELISA kits (Nanjing Jiancheng Int., Nanjing, China).

### 4.5. RT-qPCR Analysis

Total RNA was extracted from the brain stem and colon tissues using a total RNA extraction kit (DP419, Tiangen Biotech, Beijing, China). cDNA was synthesized by reverse transcription using the FastKing cDNA first chain synthesis kit (KR116, Tiangen Biotech, China), and RT-qPCR was performed using the Realab Green PCR Fast mixture kit (R0202, Lablead, Beijing, China), with GAPDH as the internal reference gene. The 2^−ΔΔCT^ method was used to process and analyze the data obtained. The primer sequences are shown in Table 1.

### 4.6. Western Blotting

Proteins in the brain and colon were extracted by RIPA lysate (WB3100, NCM, China) containing protease and phosphatase inhibitors, and then quantified by BCA protein assay kit (WB6501, NCM, Jiangsu, China). After that, the proteins were separated on an 8–10% sodium dodecyl sulfate-polyacrylamide gel (SDS-PAGE), and transferred to a polyvinylidene fluoride (PVDF) membrane, and then sealed with 5% skim milk (T1081, Solarbio, Beijing, China). The PVDF membrane was incubated with specific primary antibody at 4 °C overnight: anti-CGRP (ab139264, 1:1000, Abcam, Cambridge, UK), anti-c-Fos (ab190289, 1:1000, Abcam), anti-5-HT1A (YT4391, 1:500, Immunoway, San Jose, CA, USA), anti-5-HT3A (ab271031, 1:5000, Abcam), PKA (YT3749, 1:1000, Immunoway), PKC (YM4217, 1:500, Immunoway), Calm (YT0612, 1:1000, Immunoway), CaMKII (YT0623, 1:1000, Immunoway), P-CaMKII (YP0042, 1:1000, Immunoway), CREB (YM8342, 1:1000, Immunoway), P-CREB (28792-1-AP, 1:500, Proteintech), and anti-GAPDH (60004-lg, 1:50000, Proteintech, Hubei, China) followed by exposure to horseradish peroxidase (HRP)-coupled secondary antibodies. Then exposed to horseradish peroxidase (HRP)-coupled secondary antibodies: HRP-linked anti-mouse IgG (SA00001-1, Proteintech), HRP-linked anti-rabbit IgG (SA00001-2, Proteintech). Visualization of protein bands was conducted using enhanced chemiluminescence kit (P10200, NCM) and analyzed by ImageJ 1.5.4 software.

### 4.7. Immunofluorescence and Immunohistochemistry

Paraffin-embedded brain samples were cut into 10 μm thick sections, and the sections used for immunofluorescence experiments were treated with antibodies (anti-c-Fos, GB11069, 1:300; anti-Iba1, GB15105, 1:500, Servicebio, Hubei, China) and secondary antibodies (GB21303, 1:300; GB25301, 1:400, Servicebio) for incubation. Following DAPI counterstaining, digital images were collected using a fluorescence microscope (Nikon Eclipse C1, Tokyo, Japan). The nucleus was stained blue, Iba1 was green, and c-Fos was red. One slice from each rat was selected for staining analysis, and five fields of view were randomly selected for each slice. The average value of these five fields of view was used for the statistical analysis. Optical density was calculated using ImageJ software. The images were opened in ImageJ and converted to a grayscale image. An unstained area was selected, its average grayscale value was calculated, and this value was subtracted from the entire image. The “Measure” function in ImageJ was employed to calculate the optical density value within the image. The optical density value was normalized to the area, and statistical analysis was conducted.

The sections used in immunohistochemistry experiments were treated with an antibody (anti-AC, GB111779, 1:650, Servicebio) and a secondary antibody (GB23302, 1:300, Servicebio) for incubation. The sections were developed with 3,3′-diaminobenzidine (DAB) and then stained with hematoxylin. CaseViewer 2.4.0 was used to visualize the results, and a positive result was indicated by a brownish yellow color. The average values of the five visual fields were selected for statistical analysis. The area of positive staining was calculated using ImageJ software. The images were opened in ImageJ and the background was subtracted. An appropriate threshold was set to exclude nonspecific staining, and the “Analyze” function in ImageJ was used to calculate the percentage of the stained area and conduct statistical analysis.

### 4.8. Liquid Chromatography-Mass Spectrometry Instrumentation Analysis

The dried WZYD powder was dissolved in methanol, and the volume was adjusted to 0.2 g of raw medicine per 1 mL of solution. The solution was filtered through a 0.22 μm microporous membrane filter to obtain the filtrate. A Waters Acquity UPLC BEH C18 (2.1 × 50 mm, 1.7 μm) column was used for detection in both positive and negative ion modes on the Q Exactive HF HDMS mass spectrometer and Vanquish™Flex UHPLC Systems (Thermo, Waltham, MA, USA). Data were qualitatively analyzed using the Thermo Xcalibur 4.2 software “Qual browser”.

### 4.9. Network Pharmacology Analysis

The potential targets of the 110 chemical components identified in WZYD were predicted using the Swiss Target Prediction (http://www.swisstargetprediction.ch/ accessed on 2 July 2024) and Similarity Ensemble Approach (https://sea.bkslab.org/ accessed on 2 July 2024) databases. Using “chronic migraine” as the keyword, the Therapeutic Target Database (http://db.idrblab.net/ttd/ accessed on 2 July 2024), DisGeNET (https://www.disgenet.org/ accessed on 2 July 2024), and GeneCards (https://www.genecards.org/ accessed on 2 July 2024) databases were searched to obtain targets related to CM treatment. The targets of the WZYD potential active ingredients and the common targets of CM treatment were used for subsequent analysis. A protein−protein interaction (PPI) network was constructed using the STRING 12.0 database (https://cn.string-db.org/ accessed on 4 July 2024). Cytoscape 3.9.0 was used to construct a network diagram of the key targets and components of WZYD for CM to identify the active components related to the key targets. The MCODE plug-in was then used to cluster the key targets in the PPI network, and the key subnetwork was obtained. GO enrichment analysis was performed on the obtained targets using the DAVID (https://david.ncifcrf.gov/ accessed on 4 July 2024) database, and biological processes (BP), cellular component (CC), and molecular function (MF) related to WZYD’s treatment of CM were obtained. Through KEGG signal pathway enrichment analysis, the main pathways involved in WZYD’s treatment of CM were obtained, and the results were significant with *p* < 0.05 as the screening condition.

### 4.10. Molecular Docking

The structures of the compounds were obtained from the PubChem database (https://pubchem.ncbi.nlm.nih.gov/ accessed on 7 July 2024), while the three-dimensional structures of 5-HT1A/3A were obtained from the RCSB-PDB database (https://www.rcsb.org/ accessed on 7 July 2024). AutoDock 1.5.7 software was used to remove structural water molecules and introduce hydrogen atoms before docking, and AutoDock was used again to complete the docking of the compound with the target protein. The intensity of the interaction between the compound and protein was evaluated by analyzing the binding energy. Finally, the docking results were displayed using the PyMOL 3.1 software.

### 4.11. Data Analysis

GraphPad Prism 8 was used for the statistical analysis of the data. Firstly, the Shapiro−Wilk test was used to evaluate the normality of variance, and the data that met the normal distribution were analyzed by one-way variance, and then the Tukey test was used to analyze multiple groups. The Kruskal−Wallis test was used for data that did not meet the normal distribution, and Dunn’s test was used for multi-group analysis. Data are presented as mean ± standard deviation (SD), and *p* < 0.05 was considered statistically significant.

## 5. Conclusions

In summary, our results show that WZYD regulates the 5-HT1A and 3A receptors-mediated CREB signaling pathway by regulating 5-HT and 5-HT1A/3A receptors in the brain and colon, blocking the mutual influence between the brain and colon, and thereby reducing pain sensitization. The important role of the two receptors in the treatment of CM by WZYD was confirmed through the intervention of specific antagonists (WAY 100635) and agonists (SR 57227). This study provides experimental evidence to elucidate the unique mechanisms of action of classical TCM prescriptions. This embodies the characteristics of TCM prescriptions, which not only treat headaches but also gastrointestinal symptoms with fewer side effects.

## Figures and Tables

**Table 1:** Primer sequences for RT-qPCR.

Genes	Forward (5′-3′)	Reverse (5′-3′)
CGRP	CAGCAGAACTTGAACGCCAT	TGGATCTCAACAGCGGTCAT
c-Fos	CGGTCAAGAACATTAGCAACA	GGAACCAGACAGGTCCACAT
5-HT1A	GACCACGGCTACACCATCTA	CTTCCTGACAGTCTTGCGG
5-HT3A	GCATACCATCCAGGACATCAAC	CGTAGAACTTCATTTCCGCATAG
GAPDH	GAAGGTGAAGGTCGGAGTCAAC	CAGAGTTAAAAGCAGCCCTGGT

## Data Availability

Data is contained within the article and Supplementary Material.

## Supplementary Materials

The following supporting information can be downloaded at: https://www.mdpi.com/article/10.3390/ph18030426/s1, Figure S1: Effects of WZYD on c-Fos and Iba1 in CM rats.; Table S1: Chemical constituents of WZYD; Table S2: Molecular docking binding energy of key components with 5-HT1A/3A receptor.
