# Supplementary material for: Wuzhuyu Decoction Relieves Chronic Migraine by Regulating 5-HT1A and 3A Receptors-Mediated CREB Signaling Pathway in Brain and Intestine
Source: Pharmaceuticals (Basel). 2025 Mar 18;18(3):426. doi: 10.3390/ph18030426 (PMC11944717; doi:10.3390/ph18030426)
Supplement: Supplementary file 1 [file pharmaceuticals-18-00426-s001.zip › pharmaceuticals-3498572-supplementary.pdf]

1. Chromatographic conditions

Column temperature 35 °C; Using 0.1% formic acid water (A)-0.1% formic acid acetonitrile (B) as solvent for gradient elution: 0~5 min, 5~16% B; 5~7 min, 16~20% B; 7~11 min, 20~30% B; 11~16 min, 30~40% B; 16~21 min, 40~60% B; 21~26 min, 60~80% B; 26~29 min, 80~100% B; 29~32 min, 100~100% B; 32~32.5 min, 100~5% B; 32.5~36 min, 5~5% B. The flow rate was 0.4 $ml \cdot min^{-1}$, and the sample volume was 2 μl.

2. Effects of WZYD on c-Fos and Iba1 in CM rats

c-Fos was a key indicator of neuronal activation, while Iba1, a marker of microglial activation involved in regulating neuroinflammatory responses, both participated in the pathogenesis of chronic migraine. As shown in Fig. S1, compared with the control group, the expression of fos protein and mRNA in the model group increased significantly ($p < 0.05$). Compared with the model group, the expression of fos protein and mRNA decreased significantly after the intervention of sumatriptan, WZYD-M and WZYD-H ($p < 0.05$). The immunofluorescence results showed that, compared with the control group, the fluorescence intensity of c-Fos and Iba1 in the model group was significantly increased ($p < 0.001$); after intervention with sumatriptan, WZYD-M, and WZYD-H, compared with the model group, the fluorescence intensity of c-Fos and Iba1 was significantly decreased ($p < 0.001$). These results indicate that WZYD-M and WZYD-H can treat CM by inhibiting central neuroinflammation and the activation of nociceptive neurons.

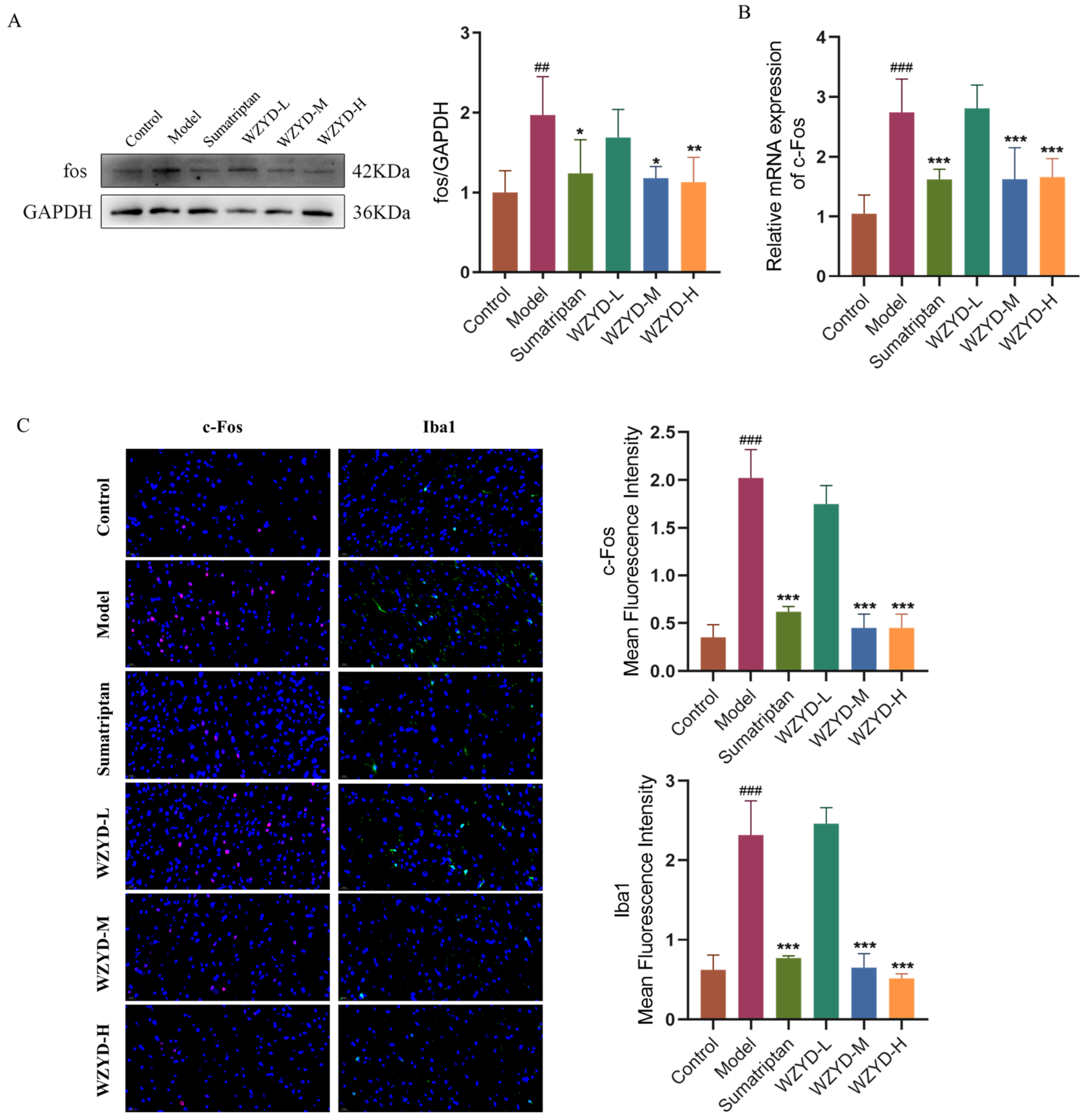

Figure S1. Effects of WZYD on c-Fos and Iba1 in CM rats. (A-B) Relative protein and mRNA expression of c-Fos in TNC (n = 5-6). (C) Relative fluorescence intensity of c-Fos and Iba1 in TNC (n = 3). Data are expressed as mean ± SD. $^{##}p < 0.01$, $^{###}p < 0.001$, vs. the control group; $^{*}p < 0.05$, $^{**}p < 0.01$, $^{***}p < 0.001$, vs. the model group.

Table S1 Chemical constituents of WZYD

No.	tR/min	Identification	Formula	$[M+H]^+$/$[M+Na]^+$	Calcd m/z	Error(ppm)	$[M-H]^-$ /$[M+COOH]^-$	Calcd m/z	Error(ppm)
1	0.7	Isocitric acid	$C_6H_8O_7$				191.0198	191.0186	6.13
2	0.91	Citric acid	$C_6H_8O_7$				191.0199	191.0186	6.65
3	1.92	Caffeoyl Gluconic acid	$C_{15}H_{18}O_{10}$	359.0948	359.0973	-6.89	357.0839	357.0816	6.38
4	2.46	Neochlorogenic acid	$C_{16}H_{18}O_9$	355.1001	355.1024	-6.36	353.0889	353.0867	6.21
5	2.51	Caffeoyl Gluconic acid	$C_{15}H_{18}O_{10}$	359.095	359.0973	-6.33	357.0836	357.0816	5.54
6	3.43	Ferulyl gluconic acid	$C_{16}H_{20}O_{10}$				371.0994	371.0973	5.73
7	3.47	Caffeoyl gluconic acid	$C_{15}H_{18}O_{10}$	359.0948	359.0973	-6.89	357.0838	357.0816	6.1
8	3.69	Chlorogenic acid	$C_{16}H_{18}O_9$	355.1	355.1024	-6.64	353.0888	353.0867	5.92
9	3.75	Catechin	$C_{15}H_{14}O_6$	291.0843	291.0863	-6.92	289.0728	289.0707	7.39
10	4.01	Cryptochlorogenic acid	$C_{16}H_{18}O_9$	355.0999	355.1024	-6.92	353.0887	353.0867	5.64
11	4.28	Ferulyl gluconic acid	$C_{16}H_{20}O_{10}$	373.1104	373.1129	-6.76	371.0995	371.0973	6
12	5.29	Trans-feruloylgluconic acid	$C_{16}H_{20}O_{10}$	373.1105	373.1129	-6.49	371.0994	371.0973	5.73
13	5.56	Feruloylquinic acid	$C_{17}H_{20}O_9$	369.1155	369.118	-6.8	367.1043	367.1024	5.29
14	6.32	Patuletin-3-Rutinoside	$C_{28}H_{32}O_{17}$	641.168	641.1712	-5.03	639.1589	639.1556	5.2
15	6.79	Quercetin 7-*O*-Rutinoside	$C_{27}H_{30}O_{16}$	611.1573	611.1607	-5.5	609.1481	609.145	5.07
16	6.95	Rutin	$C_{27}H_{30}O_{16}$	611.1572	611.1607	-5.66	609.1483	609.145	5.4
17	7.06	Hyperoside	$C_{21}H_{20}O_{12}$	465.1002	465.1028	-5.49	463.0896	463.0871	5.39
18	7.17	Kaempferol-3-*O*-Rutinoside	$C_{27}H_{30}O_{15}$	595.1627	595.1657	-5.12	593.1534	593.1501	5.57
19	7.28	Isoquercetin/Quercetin 7-Glucoside	$C_{21}H_{20}O_{12}$	465.1	465.1028	-5.92	463.0899	463.0871	6.04
20	7.33	Limocitrin 3-*O*-*β*-*D*-Xylopyranosyl(1→2)-*β*-*D*-Glucopyranoside	$C_{28}H_{32}O_{17}$	641.168	641.1712	-5.03	639.1587	639.1556	4.89
21	7.44	Isorhamnetin 3-Sambubioside	$C_{27}H_{30}O_{16}$	611.1573	611.1607	-5.5	609.1481	609.145	5.07
22	7.84	Trifolin	$C_{21}H_{20}O_{11}$	449.1053	449.1078	-5.65	447.095	447.0922	6.29
23	8.09	Limocitrin-3-*O*-Rutinoside	$C_{29}H_{34}O_{17}$	655.1833	655.1869	-5.46	653.1745	653.1712	5.01
24	8.14	Narcissin	$C_{28}H_{32}O_{16}$	625.1725	625.1763	-6.1	623.1637	623.1607	4.88

25	8.19	Astragalin	$C_{21}H_{20}O_{11}$	449.105	449.1078	-6.32	447.0948	447.0922	5.84
26	8.3	Syringetin-3-*O*-Rutinoside	$C_{29}H_{34}O_{17}$	655.1838	655.1869	-4.69	653.1747	653.1712	5.32
27	8.35	Limocitrin 3-glucoside	$C_{23}H_{24}O_{13}$	509.1261	509.129	-5.63	507.1156	507.1133	4.5
28	8.4	Isorhamnetin-3-*O*-neohesperidoside	$C_{28}H_{32}O_{16}$	625.1731	625.1763	-5.14	623.1636	623.1607	4.72
29	8.46	Chrysoeriol-7-*O*-Rutinoside	$C_{28}H_{32}O_{15}$	609.1782	609.1814	-5.25	607.1691	607.1657	5.52
30	9.32	Dehydroevodiamine	$C_{19}H_{15}N_3O$	302.1269	302.1288	-6.25			
31	9.43	Rutaecarpine-10-*O*-rutinoside	$C_{30}H_{33}N_3O_{11}$	612.2157	612.2188	-5.04			
32	9.51	20-Glucoginsenoside Rf	$C_{48}H_{82}O_{19}$	985.5316	985.5343	-2.69	1007.5463	1007.5421	4.13
33	9.73	Notoginsenoside R_1	$C_{47}H_{80}O_{18}$	955.5186	955.5237	-5.32	977.5358	977.5316	4.33
34	10.02	Ethyl-hydroxyrutaecarpine	$C_{20}H_{17}N_3O_2$	332.1374	332.1394	-5.88			
35	10.23	Ginsenoside Rg_1	$C_{42}H_{72}O_{14}$	823.4778	823.4814	-4.41	845.4932	845.4893	4.6
36	10.28	Ginsenoside Re	$C_{48}H_{82}O_{18}$	969.5346	969.5393	-4.89	991.5515	991.5472	4.32
37	11.19	Ethyl-hydroxyrutaecarpine	$C_{20}H_{17}N_3O_2$	332.1373	332.1394	-6.18			
38	11.2	Shihulimonin A	$C_{26}H_{30}O_{10}$	503.1888	503.1912	-4.72	501.1782	501.1755	5.34
39	11.52	Rutaevine Acetate	$C_{28}H_{32}O_{10}$	529.2044	529.2068	-4.58			
40	12.05	Rutaecarpine-10-*O*-*β*-*D*-glucopyranoside	$C_{24}H_{23}N_3O_7$	466.1583	466.1609	-5.53			
41	12.19	Graucin A	$C_{26}H_{30}O_{10}$	503.1884	503.1912	-5.51	501.1782	501.1755	5.34
42	13.00	3-Hydroxyrutaecarpine	$C_{18}H_{13}N_3O_2$	304.1396	304.1081	10.37			
43	13.02	Ginsenoside Rf	$C_{42}H_{72}O_{14}$	823.4771	823.4814	-5.26	845.4933	845.4893	4.72
44	13.18	Hydroxygoshuyuamide	$C_{19}H_{19}N_3O_2$	322.1529	322.155	-6.53			
45	13.42	Ginsenoside F_5	$C_{41}H_{70}O_{13}$	793.4678	793.4709	-3.86	815.4828	815.4787	4.97
46	13.53	Ginsenoside Ra_1/Ra_2	$C_{58}H_{98}O_{26}$	1233.616	1233.6239	-6.37	1255.6354	1255.6317	2.92
47	13.66	12*α*-Hydroxyrutaevin	$C_{26}H_{30}O_{10}$	503.1883	503.1912	-5.71	501.1783	501.1755	5.54
48	13.71	Ginsenoside Rb_1	$C_{54}H_{92}O_{23}$	1131.5864	1131.5927	-5.57	1153.6036	1153.6006	2.6
49	13.91	Ginsenoside Rh_1	$C_{36}H_{62}O_9$	661.4254	661.4286	-4.84	683.4396	683.4365	4.55
50	13.93	Malonylginsenoside Rb_1	$C_{57}H_{94}O_{26}$	1217.5861	1217.5926	-5.3	1193.5984	1193.595	2.88
51	14.09	Ginsenoside Rb_2/Rb_3	$C_{53}H_{90}O_{22}$	1101.5754	1101.5816	-5.62	1123.5923	1123.5895	2.51
52	14.14	Rutaevine	$C_{26}H_{30}O_9$	487.1931	487.1963	-6.48	485.183	485.1806	4.93

53	14.23	Ginsenoside F_1	$C_{36}H_{62}O_9$	661.425	661.4286	-5.45	683.4399	683.4365	4.99
54	14.29	Malonylginsenoside Rb_2/Rb_3	$C_{56}H_{92}O_{25}$	1187.5746	1187.582	-6.22	1163.589	1163.5844	3.96
55	14.3	Ginsenoside Ro	$C_{48}H_{76}O_{19}$	979.4821	979.4878	-5.82	955.4943	955.4897	4.81
56	14.52	Ginsenoside Rc	$C_{53}H_{90}O_{22}$	1101.5756	1101.5816	-5.44	1123.594	1123.5895	4.02
57	14.73	Malonylginsenoside Rc	$C_{56}H_{92}O_{25}$	1187.5771	1187.582	-4.12	1163.5889	1163.5844	3.87
58	15.1	21-OH-23-oxo-20-En-Limonin	$C_{27}H_{32}O_{10}$				515.1935	515.1912	4.52
59	15.16	Limonin	$C_{26}H_{30}O_8$	471.1986	471.2013	-5.82	469.1882	469.1857	5.34
60	15.27	14-Formyldihydrorutaecarpine	$C_{19}H_{15}N_3O_2$	318.1214	318.1237	-7.24			
61	15.32	10-Hydroxyevodiamine	$C_{19}H_{17}N_3O_2$	320.1373	320.1394	-6.41			
62	15.43	Ginsenoside Rd	$C_{48}H_{82}O_{18}$	969.5341	969.5393	-5.4	991.5519	991.5472	4.72
63	15.75	Hydroxygoshuyuamide	$C_{19}H_{19}N_3O_2$	322.1528	322.155	-6.84			
64	15.95	Evodol	$C_{26}H_{28}O_9$	485.1788	485.1806	-3.73	483.1674	483.165	5.05
65	16.39	6-Gingerol	$C_{17}H_{26}O_4$	317.1703	317.1723	-6.4			
66	16.48	7-Gingerol	$C_{18}H_{28}O_4$	309.1771	309.2060	-9.36			
67	16.5	Evodiamide	$C_{19}H_{21}N_3O$	308.1738	308.1757	-6.29			
68	16.7	Dehydrolimonin	$C_{26}H_{28}O_8$	469.1831	469.1857	-5.53	467.1728	467.17	5.9
69	16.82	6*β*-Acetoxy-5-Epilimonin	$C_{28}H_{32}O_{10}$	529.2042	529.2068	-4.96	527.1942	527.1912	5.74
70	16.99	7*β*-Acetoxy-5-Epilimonin	$C_{28}H_{32}O_{10}$	529.2041	529.2068	-5.15	527.194	527.1912	5.36
71	17.24	12*α*-Hydroxylimonin	$C_{26}H_{30}O_9$				485.1832	485.1806	5.34
72	17.25	Evodiamine	$C_{19}H_{17}N_3O$	304.1425	304.1444	-6.37			
73	17.31	Rutaevine Acetate	$C_{28}H_{32}O_{10}$	529.204	529.2068	-5.34	527.1938	527.1912	4.98
74	17.68	Rutaecarpine	$C_{18}H_{13}N_3O$	288.1113	288.1131	-6.38			
75	17.79	3-Hydroxyevodiamine	$C_{19}H_{17}N_3O_2$	320.1374	320.1394	-6.1			
76	17.94	Ginsenoside F_4	$C_{42}H_{70}O_{12}$				811.4893	811.4838	6.74
77	18.16	Obacunone	$C_{26}H_{30}O_7$	455.2038	455.2064	-5.78			
78	18.64	1-Methyl-2-[Hydroxyl-Tridecenyl]-4(1H)-Quinolone	$C_{23}H_{35}NO_2$	358.2715	358.2741	-7.13			
79	18.81	1-Methyl-2-[Hydroxyl-Tridecenyl]-4(1H)-Quinolone	$C_{23}H_{35}NO_2$	358.2719	358.2741	-6.02			

80	18.91	Goshuyuamide I	$C_{19}H_{19}N_3O$	306.1579	306.1601	-7.15			
81	18.97	1-Methyl-2-[Hydroxyl-Tridecenyl]-4(1H)-Quinolone	$C_{23}H_{35}NO_2$	358.2715	358.2741	-7.13			
82	19.02	1-Methyl-2-Nonadecyl-4(1H)-Quinolone	$C_{19}H_{25}NO$	284.199	284.2009	-6.65			
83	19.43	Ginsenoside F_2	$C_{42}H_{72}O_{13}$	807.4822	807.4865	-5.34	829.4989	829.4944	5.43
84	20.57	1-Methyl-2-Nonyl-4(1H)-quinolone	$C_{19}H_{27}NO$	286.2145	286.2165	-7.13			
85	20.66	4-Gingerol	$C_{15}H_{22}O_4$				265.1487	265.1434	-6.21
86	20.89	6-Shogaol	$C_{17}H_{24}O_3$	277.1781	277.1798	-6.21			
87	20.95	1-Methyl-2-[tetradecenyl]-4(1H)-quinolone	$C_{23}H_{27}NO$	334.2144	334.2165	-6.41			
88	21.48	1-Methy1-2-[(*Z*)-5-undecenyl]-4(1H)-quinolone	$C_{21}H_{29}NO$	312.2302	312.2322	-6.38			
89	21.59	1-Methyl-2-[tetradecatrienyl]-4(1H)-quinolone	$C_{23}H_{29}NO$	336.23	336.2322	-6.52			
90	21.85	1-Methyl-2-Decyl-4(1H)-Quinolone	$C_{20}H_{29}NO$	300.2303	300.2322	-6.3			
91	21.95	Ginsenoside Rk_1	$C_{42}H_{70}O_{12}$				811.4877	811.4838	4.77
92	22.23	[6]-Dehydrogingerdione	$C_{17}H_{22}O_4$	291.1572	291.1591	-0.65			
93	22.28	1-Methy1-2-[(*Z*)-5'-dodecenyl]-4(1H)-quinolone	$C_{22}H_{31}NO$	326.2455	326.2478	-7.18			
94	22.55	1-Methyl-2-[-pentadecatetraenyl]-4(1H)-quinolone	$C_{25}H_{31}NO$	362.2455	362.2478	-6.46			
95	22.55	6-Gingediacetate	$C_{21}H_{32}O_6$	363.2487	363.2166	8.84			
96	22.72	8-Gingerol	$C_{19}H_{30}O_4$	323.2194	323.2217	-7.07	321.2082	321.206	6.74
97	22.82	1-Methyl-2-[-pentadecatetraenyl]-4(1H)-quinolone	$C_{25}H_{31}NO$	362.2452	362.2478	-7.29			
98	22.88	10-Gingerol	$C_{21}H_{34}O_4$	373.2323	373.2349	-7.05			
99	23.09	1-Methyl-2-Undecyl-4(1H)-Quinolone	$C_{21}H_{31}NO$	314.2457	314.2478	-6.81			
100	23.2	1-Methyl-2-[pentadecatrienyl]-4(1H)-quinolone	$C_{25}H_{33}NO$	364.2611	364.2635	-6.56			
101	23.52	8-Gingerdiol	$C_{19}H_{32}O_4$	325.235	325.2373	-7.18	323.224	323.2217	7.16

102	23.68	Evocarpine	$C_{23}H_{33}NO$	340.2612	340.2635	-6.73			
103	24.27	1-Methyl-2-Dodecyl-4(1H)-Quinolone	$C_{22}H_{33}NO$	328.2614	328.2635	-6.37			
104	24.43	1-Methy1-2-[(6*Z*,9*Z*)-6,9-pentadecadienyl]-4(1H)-quinolone	$C_{25}H_{35}NO$	366.2768	366.2791	-6.39			
105	25.15	Oleanolic acid/Betulinic acid	$C_{30}H_{48}O_3$				455.3548	455.352	6.21
106	25.39	Dihydroevocarpine	$C_{23}H_{35}NO$	342.2768	342.2791	-6.84			
107	25.82	1-Methyl-2-[Pentadecenyl]-4(1H)-Quinolone	$C_{25}H_{37}NO$	368.2923	368.2948	-6.76			
108	26.47	1-Methyl-2-Tetradecyl-4(1H)-Quinolone	$C_{24}H_{37}NO$	356.2924	356.2948	-6.71			
109	27.49	1-Methyl-2-pentadecyl-4(1H)-quinolone	$C_{25}H_{39}NO$	370.3082	370.3104	-6.05			
110	29.10	Protopanaxatriol	$C_{30}H_{52}O_4$	477.3889	477.3938	-1.03			

Table S2 Molecular docking binding energy of key components with 5-HT1A/3A receptor

Compounds	binding energy	
	5-HT 1A	5-HT 3A
3-Hydroxyevodiamine	-8.947	-9.904
3-Hydroxyrutaecarpine	-9.596	-10.07
10-Hydroxyevodiamine	-8.731	-9.093
1-Methyl-2-[(6*Z*,9*Z*)-69-pentadecadienyl]-4(1H)-quinolone	-7.373	-8.425
1-Methyl-2-[(*Z*)-5-undecenyl]-4(1H)-quinolone	-7.802	-7.994
1-Methyl-2-[(*Z*)-5'-dodecenyl]-4(1H)-quinolone	-7.902	-8.07
14-Formyldihydrorutaecarpine	-8.442	-9.634

Trans-feruloylgluconic acid	-7.309	-6.927
Goshuyuamide I	-8.469	-8.94
Dehydroevodiamine	-9.909	-9.909
Evodiamine	-8.76	-8.434
Protopanaxatriol	-9.023	-8.839
Limonin	-9.643	-8.812
[6]-Dehydrogingerdione	-7.319	-8.047
Evocarpine	-7.315	-7.694
4-Gingerol	-7.255	-7.454
6-Gingerol	-6.902	-7.021
6-Shogaol	-7.767	-7.889
6-Gingediacetate	-7.5	-7.709
7-Gingerol	-7.506	-6.927
8-Gingerol	-7.053	-8.019
8-Gingerdiol	-6.83	-7.625
10-Gingerol	-6.975	-7.379
